# Metal–Organic Framework-Enabled Trapping of Volatile Organic Compounds into Plasmonic Nanogaps for Surface-Enhanced Raman Scattering Detection

**DOI:** 10.1021/acsnano.4c00208

**Published:** 2024-04-17

**Authors:** Yi Liu, Ka Kit Chui, Yini Fang, Shizheng Wen, Xiaolu Zhuo, Jianfang Wang

**Affiliations:** † Department of Physics, The Chinese University of Hong Kong, Shatin, Hong Kong SAR 999077, China; ‡ Jiangsu Province Key Laboratory of Modern Measurement Technology and Intelligent Systems, School of Physics and Electronic Electrical Engineering, Huaiyin Normal University, Huaian 223300, China; § School of Science and Engineering, The Chinese University of Hong Kong (Shenzhen), Shenzhen 518172, China

**Keywords:** gold nanocrystals, metal−organic frameworks, particle-on-mirror structures, plasmon coupling, plasmon resonance, surface-enhanced Raman scattering

## Abstract

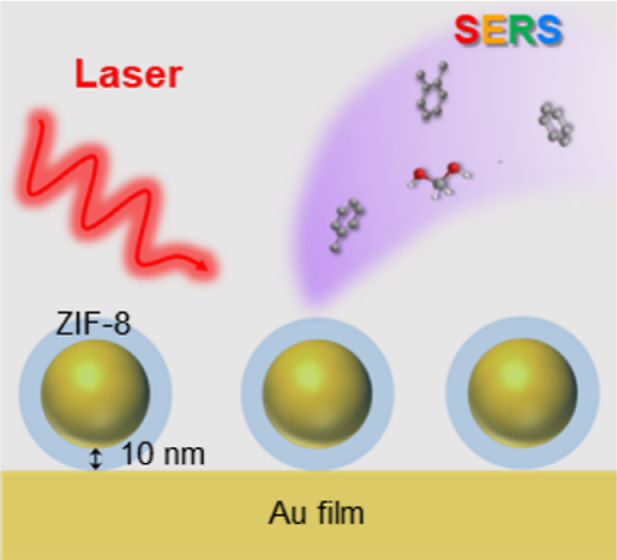

Utilizing electromagnetic hotspots within plasmonic nanogaps is a promising approach to create ultrasensitive surface-enhanced Raman scattering (SERS) substrates. However, it is difficult for many molecules to get positioned in such nanogaps. Metal–organic frameworks (MOFs) are commonly used to absorb and concentrate diverse molecules. Herein, we combine these two strategies by introducing MOFs into plasmon-coupled nanogaps, which has so far remained experimentally challenging. Ultrasensitive SERS substrates are fabricated through the construction of nanoparticle-on-mirror structures, where Au nanocrystals are encapsulated with a zeolitic imidazolate framework-8 (ZIF-8) shell and then coupled to a gold film. The ZIF-8 shell, as a spacer that separates the Au nanocrystal and the Au film, can be adjusted in thickness over a wide range, which allows the electric field enhancement and plasmon resonance wavelength to be varied. By trapping Raman-active molecules within the ZIF-8 shell, we show that our plasmon-coupled structures exhibit a superior SERS detection performance. A range of volatile organic compounds at the concentrations of 10^–2^ mg m^–3^ can be detected sensitively and reliably. Our study therefore offers an attractive route for synergistically combining plasmonic electric field enhancement and MOF-enabled molecular enrichment to design and create SERS substrates for ultrasensitive detection.

Surface-enhanced Raman scattering (SERS) has found wide applications in bioanalysis, environment, food safety, sensing, and catalysis.^1−4^ One key factor for outstanding SERS performance is the plasmonic substrate.^5,6^ Noble metal nanostructures of various shapes and architectures have been studied and used as SERS substrates. Substrates that provide nanogaps among neighboring metal nanoparticles can often give rise to the strongest SERS signals because the electric field in the nanogaps can be greatly enhanced by ∼10^2^–10^5^ folds.^7,8^ Such nanogaps are known as electromagnetic hotspots. Moreover, most SERS studies and applications have relied on molecules that can bind strongly to the noble metal surface, such as thiol- and amino-containing molecules.^9^ However, there exist many more molecules that can adsorb poorly only on the noble metal surface. These molecules, such as volatile organic compounds (VOCs), usually exhibit low SERS sensitivities. Many such molecules can be frequently found in our living environment but are harmful to humans. It is necessary and urgent to detect such poorly adsorbed molecules. Furthermore, hotspots in the nanogaps are usually on the scale of a few nanometers.^10,11^ It is often extremely challenging to position nonadsorbing molecules in the nanoscale hotspots.

The integration of metal–organic frameworks (MOFs) with noble metal nanocrystals overcomes these limitations. MOFs are a class of highly porous crystalline material composed of inorganic metal nodes and organic linkers.^12^ Because of their unique three-dimensional open networks, high nanoscale porosities, and tunable structures,^13,14^ MOFs can trap molecules that do not possess specific metal affinities into their pores and cavities.^15,16^ Strategies based on the combination of MOFs and plasmonic metals have been reported to enable a reliable and sensitive SERS analysis. For example, NU-901,^17,18^ MIL-101,^19^ and ZIF-8^20−22^ have been successfully coated on diverse plasmonic nanocrystals, such as Au nanospheres (NSs), nanorods (NRs), and Ag nanocubes, giving rise to core@shell structures, so as to enrich target molecules around the surfaces of plasmonic nanocrystals for SERS measurements. Layer-by-layer structures have also been fabricated by depositing MOF layers on plasmonic nanocrystal arrays.^23,24^ Heterostructures with ZIF-8 selectively deposited at strong electric field enhancement sites have also been designed to concentrate analyte molecules into hotspots.^25^ However, the thick ZIF-8 layers of more than 100 nm in previous studies can impede the diffusion of target molecules from the outside to the plasmonic nanoparticle surface, consequently stifling the Raman signal. In addition, these previous works have merely demonstrated the use of hotspots on individual plasmonic nanocrystals, which suffer from lower electric field enhancement in comparison with plasmonic nanogaps. The introduction of MOFs into hotspots that are formed from plasmonic nanogaps has not been realized, as it has remained challenging to synthesize MOFs with their sizes as small as a few nanometers. It is this challenge that our study seeks to overcome.

In this work, we demonstrate a simple and general approach to position ZIF-8 (simplified as ZIF below) into plasmonic nanogap hotspots by forming nanoparticle-on-mirror structures. ZIF is first coated on the entire surface of Au NSs to give (Au NS core)@(ZIF shell) nanoparticles, which are then drop-cast on a gold film to fabricate plasmon-coupled SERS substrates. The gap distance, as well as the plasmon-coupling strength between the Au NSs and the Au film, is highly adjustable by varying the ZIF thickness, which can be controlled precisely in the range of 3–60 nm (Figure 1). The successful coating of the ZIF shell onto anisotropic Au NRs and hexagonal Au nanoplates (Au NPLs) further firmly demonstrates the generality of this approach. We studied the plasmon resonance behaviors of the nanoparticle-on-mirror structures by single-particle dark-field scattering measurements and investigated the impacts of the different parameters on their SERS performance. Our plasmon-coupled structures exhibit superior SERS performance because of the combined contributions of the hotspots in the plasmonic nanogaps and the ZIF-assisted molecular trapping effect. The detection capabilities of the as-prepared SERS substrates satisfy the requirements of indoor air quality monitoring. Our work combines the advantages of the plasmonic nanogap hotspots and the molecular enrichment effect for SERS detection, providing a facile yet encouraging route to the fabrication of SERS substrates with great potential for ultrasensitive and quantitative detection of environmental pollutants.

**Figure 1:**
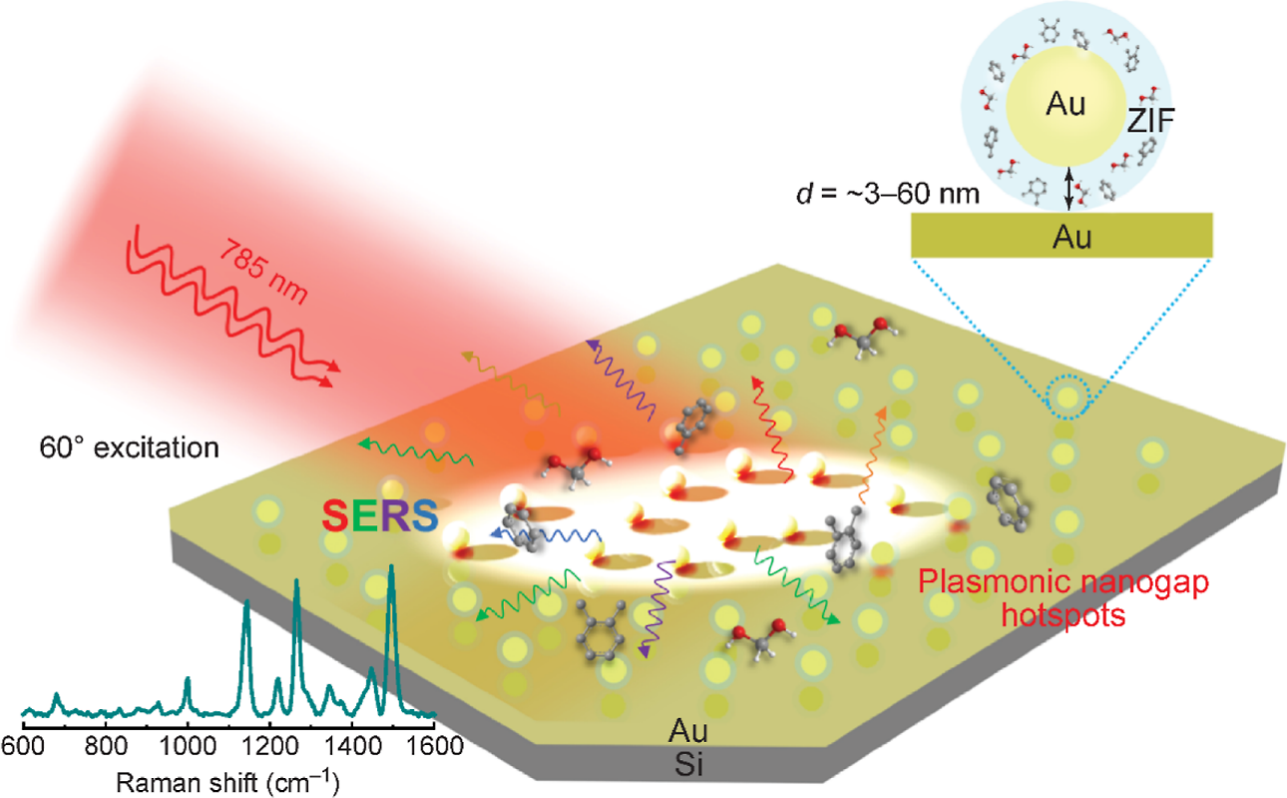
Schematic illustration of the plasmon-coupled SERS substrate. The nanogap hotspots are filled with ZIF for trapping organic molecules.

## Results and Discussion

Au NSs with an average diameter of 92.0 ± 3.1 nm were first grown as the plasmonic cores.^26^ Among various MOFs, ZIF constructed from methylimidazole (Hmim) and zinc salt (Zn^2+^) was employed as the porous shell because of its mild preparation conditions.^25^ During the synthesis of (Au NS)@ZIF nanoparticles, the cetyltrimethylammonium bromide (CTAB) surfactant plays a dual role in the stabilization of the Au NSs and the regulation of the ZIF size. The hydrophobic carbon chain of CTAB has been reported to adsorb preferentially on the {100} facets of ZIF nanocrystals to minimize the surface energy and suppress the ZIF crystallization.^27,28^ In general, the ZIF thickness is controlled by the CTAB concentration, concentrations of the ZIF precursors, and addition sequence of the ZIF precursors. As the concentration of CTAB was increased from 0 to 161 μM, while the amounts of the ZIF precursors and the Au NSs were kept unchanged, the average ZIF thickness of the final product was reduced from 10.6 to 3.5 nm (Figures S1 and S2). The measured extinction spectra show that the plasmon resonance peaks of the as-prepared NS@ZIF samples are slightly red-shifted with the increase in the ZIF shell thickness (Figure S3), which is caused by the higher refractive index of ZIF (∼1.42) than that of water (1.33).^21,29^ With the CTAB concentration kept at 46 μM, the ZIF shell thickness was reduced from 12.5 to 7.8 nm as the concentrations of Hmim/Zn^2+^ were reduced from 1.056 M/19.2 mM to 0.792 M/14.4 mM (Figures S4–S6). The thickness reduction can be attributed to the reduced nucleation and growth rates of the ZIF nanocrystals on the surface of the Au NSs. When the concentrations of Hmim and Zn^2+^ were further lowered to 0.528 M and 9.6 mM, the nucleation and growth of ZIF encountered large hindrance.^25,27^ Small ZIF nanocrystals were formed and randomly deposited on the surface of the Au NSs, leading to the formation of a discontinuous shell (Figure S4e). Interestingly, a thicker ZIF shell was obtained when the Zn^2+^ solution was added before the Hmim solution (Figures S7–S9). A majority of Hmim molecules are available to reach the Au surface and engage with the adsorbed Zn^2+^ ions, consequently promoting the growth rate of the ZIF shell. This phenomenon is associated with the difference in the diffusion barrier between Hmim molecules and Zn^2+^ ions in the reaction solution.

**Figure 2:**
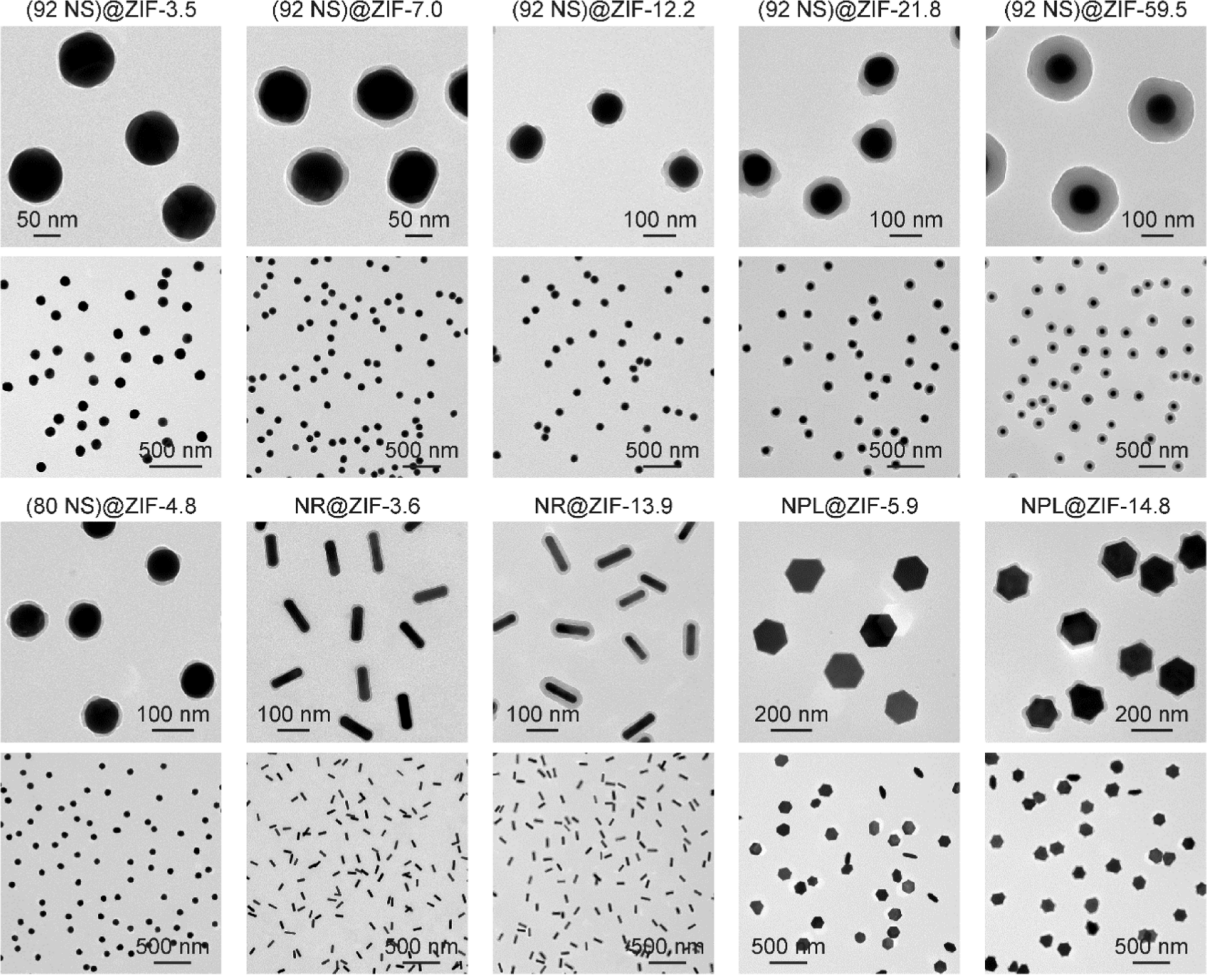
TEM images of the (92 NS)@ZIF, (80 NS)@ZIF, NR@ZIF, and NPL@ZIF nanoparticles with different shell thicknesses under high and low magnifications.

The core@shell nanoparticles composed of the Au NSs with an average diameter of 92 nm and ZIF shell with an average thickness of *x* nm are denoted as (92 NS)@ZIF-*x*. TEM imaging reveals that the (92 NS)@ZIF nanoparticles all have narrow size distributions in the ZIF shell thickness (Figure 2), demonstrating the high robustness of our method. The X-ray diffraction (XRD) patterns indicate that the Au nanoparticles and the ZIF shell are highly crystalline (Figure S10). The presence of the C, N, and Zn elements was confirmed by energy-dispersive X-ray analysis (Figure S11). Furthermore, high-angle annular dark-field scanning transmission electron microscopy imaging and elemental mapping were performed on the individual NS@ZIF nanoparticles (Figure S12). The Au NS core is preserved, and the Zn, C, and N elements from the ZIF shell are evenly distributed around the Au NS, confirming again the structural integrity of the NS@ZIF nanoparticles. More importantly, we successfully extended this synthetic strategy to Au nanocrystals with different sizes and shapes, such as smaller Au NSs, anisotropic Au NRs, and hexagonal Au NPLs (Methods in the Supporting Information). The resultant (Au core)@(ZIF shell) nanoparticles are named (80 NS)@ZIF, NR@ZIF, and NPL@ZIF, respectively (Figures 2 and S13–S21). In addition to shapes and sizes, this method is also applicable for nanoparticles of different compositions such as Ag nanocubes and Ag nanorods (Figure S22). All the core@shell nanoparticle samples consistently exhibit great uniformity and dispersibility, indicating that this approach of tailoring the ZIF thickness in the nanometer range is applicable to various metal nanocrystals.

To construct the nanoparticle-on-mirror structures, we deposited 100 nm thick Au films on smooth Si substrates, together with a 5 nm thick Ti adhesion layer, by electron-beam evaporation. According to previous studies of nanoparticle-on-mirror systems,^30,31^ the Au film in our system is thick enough to generate intensive electric field enhancement when it is coupled with the Au NSs. The prepared (Au core)@(ZIF shell) nanoparticles with different ZIF thicknesses were dispersed in methanol and drop-cast on the Au films. The gap distance between the bottom of the Au nanocrystals and the surface of the Au film is controlled by the ZIF thickness. When the ZIF thickness is small, that is, when the Au nanocrystals are very close to the Au film, the charge distribution in the Au film is disturbed, leading to the formation of image charges in the Au film induced by the original charges on the Au nanocrystals. A robust interaction between the original charges and the image charges results in the coupling of their plasmon resonances, thereby greatly enhancing the electric field within the nanogaps. As a result, Au-film-coupled (Au core)@(ZIF shell) nanoparticles with adjustable gap distances and electric field enhancements were successfully prepared, and the porous ZIF was favorably positioned into the plasmon-coupled nanogaps.

**Figure 3:**
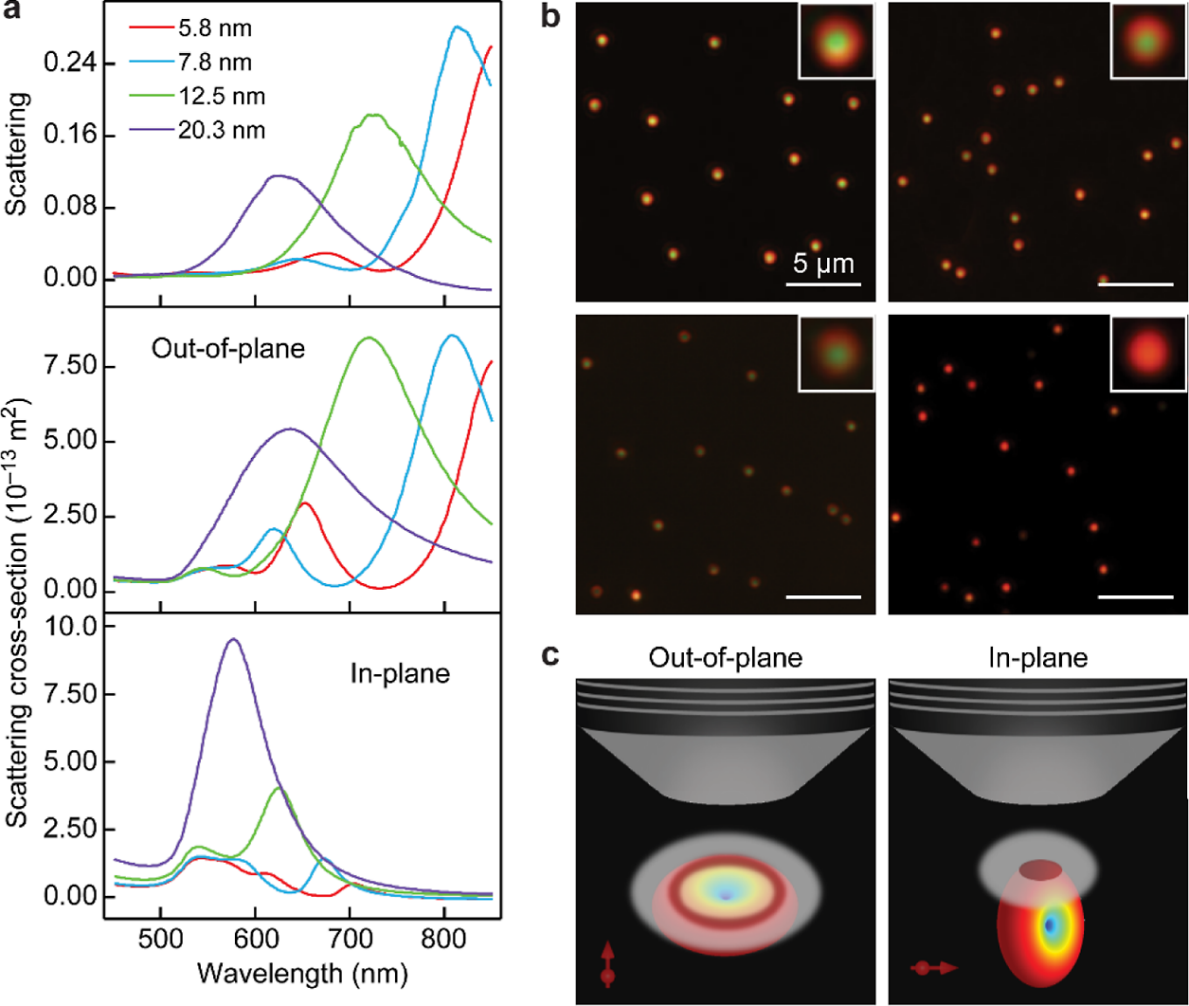
Plasmonic properties of the (92 NS)@ZIF/film structures with different ZIF thicknesses. (a) Single-particle dark-field scattering spectra. Upper: experimental scattering spectra; middle: simulated scattering spectra under out-of-plane excitation; and bottom: simulated scattering spectra under in-plane excitation. (b) Dark-field scattering images. The gap distances are 5.8 (top left), 7.8 (top right), 12.5 (bottom left), and 20.3 nm (bottom right), respectively. (c) Schematics of the far-field scattering patterns under the out-of-plane (left) and in-plane (right) excitation polarization directions relative to the Au film. The red arrows indicate the oriented dipoles. The gray plates on the radiation torus indicate the focal plane of the objective.

In order to investigate the optical response of the plasmon-coupled structures, we performed single-particle dark-field scattering measurements on (92 NS)@ZIF nanoparticles deposited on the Au films. Four samples with different ZIF thicknesses (5.8, 7.8, 12.5, and 20.3 nm) were prepared and used to examine the effect of the gap distance on the plasmon resonance wavelength of the plasmon-coupled structures. The surface area coverage of the NS@ZIF nanoparticles on the Au film was intentionally kept low (∼4 nanoparticles per 100 μm^2^) to facilitate the single-particle scattering measurements. The scattering spectra of 15 randomly selected NS@ZIF nanoparticles were recorded and subsequently averaged. Regardless of the ZIF thickness, all the scattering spectra display a dominant plasmon band in the red to near-infrared region, which blue-shifts with increasing ZIF thicknesses (Figure 3a upper and Table S1). The shifting plasmon band is also accompanied by variation of the single-particle dark-field images (Figure 3b). When the ZIF thickness is thin (5.8 nm), the dark-field image appears as a doughnut shape in red color with a green spot at the center. As the ZIF thickness is increased, the dark-field scattering images gradually transform into solid red spots. The blueshift of the dipole plasmon mode, along with the reshaping of the far-field scattering pattern from the doughnut shape to a solid spot, intuitively indicates a reduction in the plasmon coupling strength as the gap distance is increased. Moreover, the monodispersity of our samples is further evidenced by the uniform dark-field scattering patterns over a relatively large area.

To further understand the above experimental observations, we carried out finite-difference time-domain (FDTD) simulations on the (92 NS)@ZIF/film structures with different ZIF thicknesses. FDTD-simulated extinction spectra were first performed, which are in good agreement with the experimentally measured spectra in the shape and peak position (Figure S23), demonstrating the effectiveness of our parameter setting. Two excitation schemes, where the electric field polarization was either perpendicular or parallel to the Au film, were thereafter considered to simulate the out-of-plane and in-plane plasmon resonance modes, respectively. Under the out-of-plane excitation configuration, the simulated spectra exhibit a major plasmon band that blueshifts as the ZIF coating becomes thicker (Figure 3a, middle), which matches well with the experimental scattering spectra. This plasmon band is typically associated with a vertically coupled mode supported by the nanoparticle-on-mirror system, giving rise to a doughnut-shaped pattern in the far-field scattering image (Figure 3c).^32,33^ At the same time, a weak plasmon peak was observed in the shorter-wavelength region. It can be ascribed to a quadrupole plasmon mode, which gives less contribution to the electric field enhancement. In the case of in-plane excitation, a blueshift of the simulated plasmon band was also observed with the increase in the ZIF thickness (Figure 3a, bottom). The in-plane plasmon mode typically presents a solid spot in the far-field scattering image (Figure 3c). When the ZIF thickness is thin, it is difficult to distinguish this in-plane peak in the experimental spectra because of the cancellation between the original dipole and the image dipole and the overlap of the in-plane mode with the strong out-of-plane mode.^34,35^ The in-plane mode is, however, distinguishable as a central green spot in the dark-field scattering images. Taken together, the experimental dark-field images are a combination of the far-field scattering patterns of the in-plane and out-of-plane plasmon modes. When the gap distance is sufficiently small, the scattered light is dominantly caused by the out-of-plane plasmon mode, indicating strong plasmon coupling between the Au NS and the Au film. As the ZIF shell gets very thick, such as 20.3 nm in our structure, the large gap distance greatly weakens the dipole interaction between the Au NS and the Au film.^36,37^ The weak plasmon coupling effect eventually leads to a bright red solid spot occurring in the far-field scattering image.

The Au NSs with an average diameter of 80 nm were also employed to examine the distance-dependent plasmon coupling and to investigate their plasmon resonance wavelength variation. We repeated the same measurements with the (80 NS)@ZIF/film structures and observed similar spectral changes and dark-field scattering images (Figure S24 and Table S1). However, the wavelength and line width of the plasmon band are different from those of the (92 NS)@ZIF/film structures because the plasmon coupling strength between the Au NS and the Au film is also dependent on the Au NS size. One can expect more possibilities when replacing the (Au NS)@ZIF nanoparticles with the (Au NR)@ZIF and (Au NPL)@ZIF nanoparticles, whose dimensional parameters enable a wide range of tunability for the coupled system, not only in the plasmon wavelength and line width but also in the polarization and contact area.^38^ In the discussion below, we mainly focus on the (Au NS)@ZIF/film structures, as a proof-of-principle, to demonstrate the superiority of the SERS substrates based on this nanoparticle-on-mirror system.

**Figure 4:**
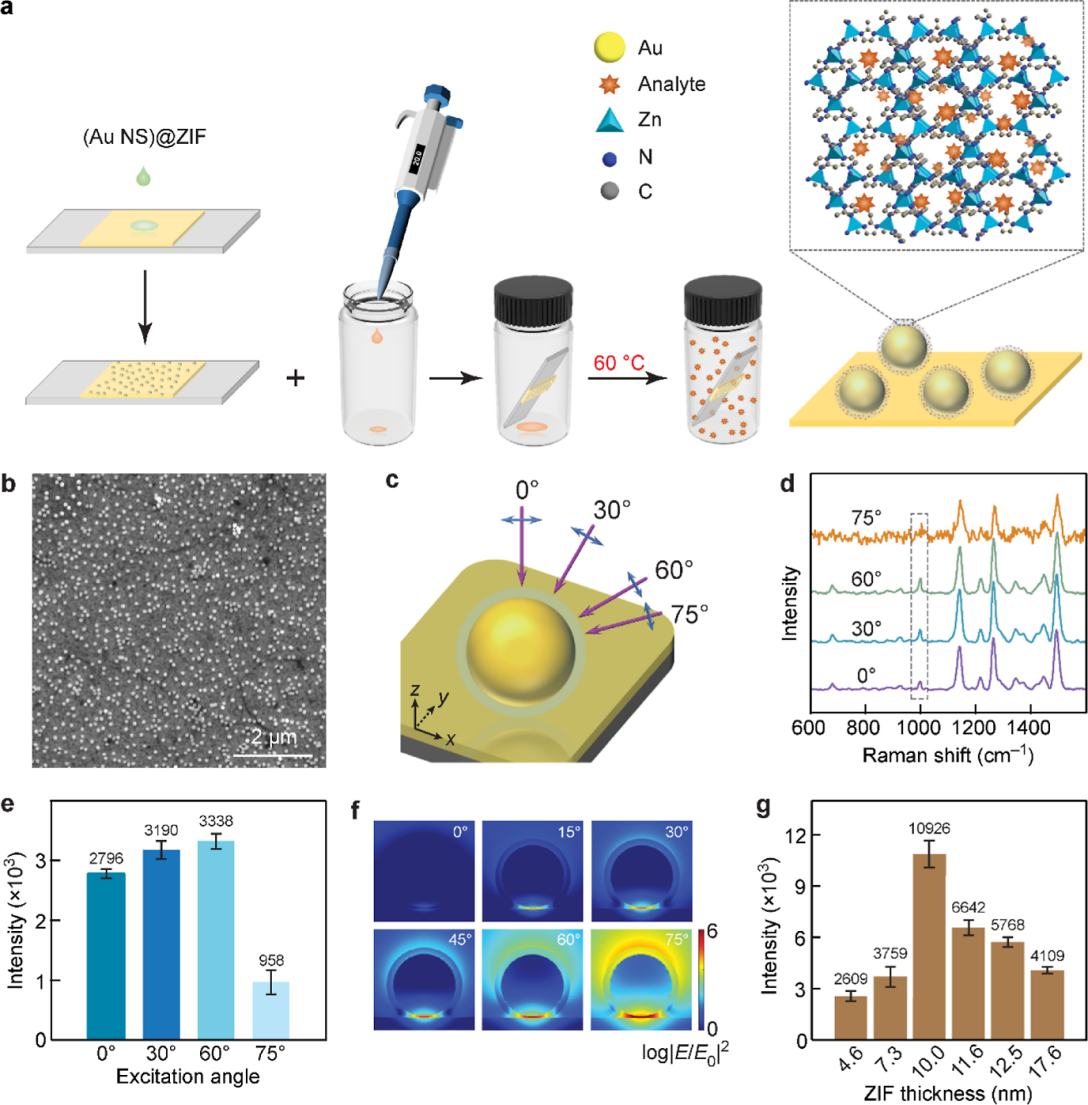
Molecular trapping and SERS activity of the NS@ZIF/film structures. (a) Schematic illustrating the diffusion of the gaseous analyte molecules into the porous ZIF shell. (b) SEM image of the (92 NS)@ZIF nanoparticles uniformly dispersed on the Au film. (c) Schematic of the different incidence angles of the excitation laser light relative to the normal of the Au film. The purple arrows indicate the wavevector of the excitation light. The blue double-headed arrows refer to the polarization direction of the excitation light. (d) SERS spectra of toluene at different excitation angles. The dashed box highlights the characteristic peak of toluene. The (92 NS)@ZIF/film structures with a gap distance of 8.3 nm were used as the SERS substrate. The concentration of toluene is 2.89 × 10^4^ mg m^–3^. (e) Integrated intensities of the characteristic peak of toluene indicated with the dashed box in (d). (f) Simulated electric field intensity enhancement contours at the plasmon-coupled nanogaps. The excitation wavelength is 785 nm. (g) Dependence of the SERS intensities on the shell thickness. The (92 NS)@ZIF/film structures with varying gap distances were used as the SERS substrates for the detection of toluene at a concentration of 2.89 × 10^7^ mg m^–3^.

SERS enhancement has been reported to be strongly correlated with the plasmon wavelength of the SERS substrate. The maximal SERS intensity can be achieved when the plasmon resonance wavelength is approximately at the center between the excitation wavelength and the Raman emission wavelength of the probe molecule.^39,40^ In our study, a portable Raman spectrometer with a fixed excitation wavelength of 785 nm was employed. We tailored the plasmon resonance of the NS@ZIF/film substrate according to the emission wavelengths of the target molecules. As shown in Figures 3a and S24, the plasmon resonance is adjusted by modifying the ZIF shell thickness and the diameter of the plasmonic nanoparticle. From the comprehensive analysis of Au nanoparticles of various shapes and sizes, we found that Au NSs with diameters of 80 and 92 nm yielded an ideal plasmon resonance near 818 nm when paired with the appropriately tuned shell thickness, as detailed in Table S1. The (92 NS)@ZIF/film structures were employed tentatively to investigate the SERS activity in the detection of toluene. The trapping of the gaseous analyte molecules in the plasmonic nanogaps was accomplished in a sealed vial (Figure 4a). The prepared NS@ZIF nanoparticles in methanol were drop-cast on the Au film. Due to the low surface tension of the methanol droplets, the NS@ZIF nanoparticles were distributed uniformly on the Au film (Figure 4b), which minimized the fluctuation of the SERS signals originating from the coffee-ring effect and thus the interparticle plasmon coupling effect. The concentration of the NS@ZIF nanoparticles on the Au film was ∼20 nanoparticles per μm^2^ and was kept the same for all SERS measurements. The intensity of the electric field in the plasmonic nanogaps strongly relies on the direction of the excitation electric field.^41,42^ The effect of the incidence angle of the excitation laser light on the SERS performance of the NS@ZIF/film structure was therefore investigated. The incidence angle in the SERS measurement was changed from 0 to 75° with respect to the normal of the Au film, causing the polarization direction to vary from in-plane to nearly out-of-plane (Figure 4c). The experimental result is shown in Figure 4d. Toluene exhibits a characteristic peak at 1000 cm^–1^. The peaks at 682, 1134, 1360, and 1495 cm^–1^ belong to the ZIF shell. The integral analysis of the 1000 cm^–1^ peak indicates that the maximal SERS intensity is obtained when the incidence angle of the excitation laser light is 60° (Figure 4e). It is worth noting that a relatively strong SERS intensity is also collected at normal incidence. Since the original dipole is nearly canceled by the induced image dipole under in-plane-polarized excitation, the Raman enhancement in this case should be very small. The experimentally obtained large SERS signal at normal incidence can therefore be ascribed to the diffuse scattering by the Au-film-coupled NS@ZIF nanoparticles. Furthermore, FDTD simulations confirmed that the electric field intensity in the plasmonic nanogap increases as the incidence angle of the excitation light is enlarged (Figures 4f and S25). When the excitation angle is adjusted to 75°, 93% of the incidence light is in the out-of-plane polarization, which results in a strong electric field enhancement between the Au NS and the Au film. However, the SERS signal obtained in the experiment is not as strong as expected. This can be attributed to the non-uniform spatial distribution of the Raman scattering.^43,44^ The Raman scattering signal collected by the portable detector is relatively weak at 75°, which was also verified by the low signal-to-noise ratio in Figure 4d. Therefore, the best SERS performance of our NS@ZIF/film structure is achieved at the 60° incidence angle owing to a joint action of the excitation polarization and the collection of the Raman scattering signal. The subsequent SERS measurements were all performed at an excitation angle of 60°.

The plasmonic gap distance is controlled by the ZIF thickness and plays a vital role in SERS performance. The enlargement of the shell thickness reduces the electric field enhancement and results in a low Raman enhancement factor.^45,46^ In the absence of any target analyte, the SERS intensity originating from the ZIF shell becomes weaker with the increase in the shell thickness (Figure S26), illustrating the distance-dependent SERS performance. However, the SERS results on toluene detection show that the SERS intensity first increases and then decreases with thickening of the ZIF shell (Figures 4g and S27). The experimental result is not in line with the expectation that the smaller the gap distance, the larger the electric field enhancement and the better the SERS performance. The experimental result illustrates that the SERS performance is not solely affected by the electric field enhancement in the hotspot but is also dependent on the enrichment effect of the ZIF shell.^22,23^ When the ZIF shell is thin, the enrichment effect of the ZIF shell on the vapor molecules is limited and the number of molecules trapped in the plasmonic nanogap hotspot is relatively small. As the ZIF shell becomes thicker, more target molecules can be trapped at the hotspot and have their Raman signals enhanced. With a further increase in the ZIF thickness, the plasmon coupling becomes weak and the electric field enhancement becomes smaller, resulting in a decrease in the SERS intensity. As a result, the interplay between the electric field enhancement and the enrichment effect of the ZIF shell enables the best SERS performance to be achieved at a ZIF thickness of ∼10 nm. This conclusion was further confirmed by the SERS measurements using the (80 NS)@ZIF/film structures as the SERS substrates (Figure S28).

**Figure 5:**
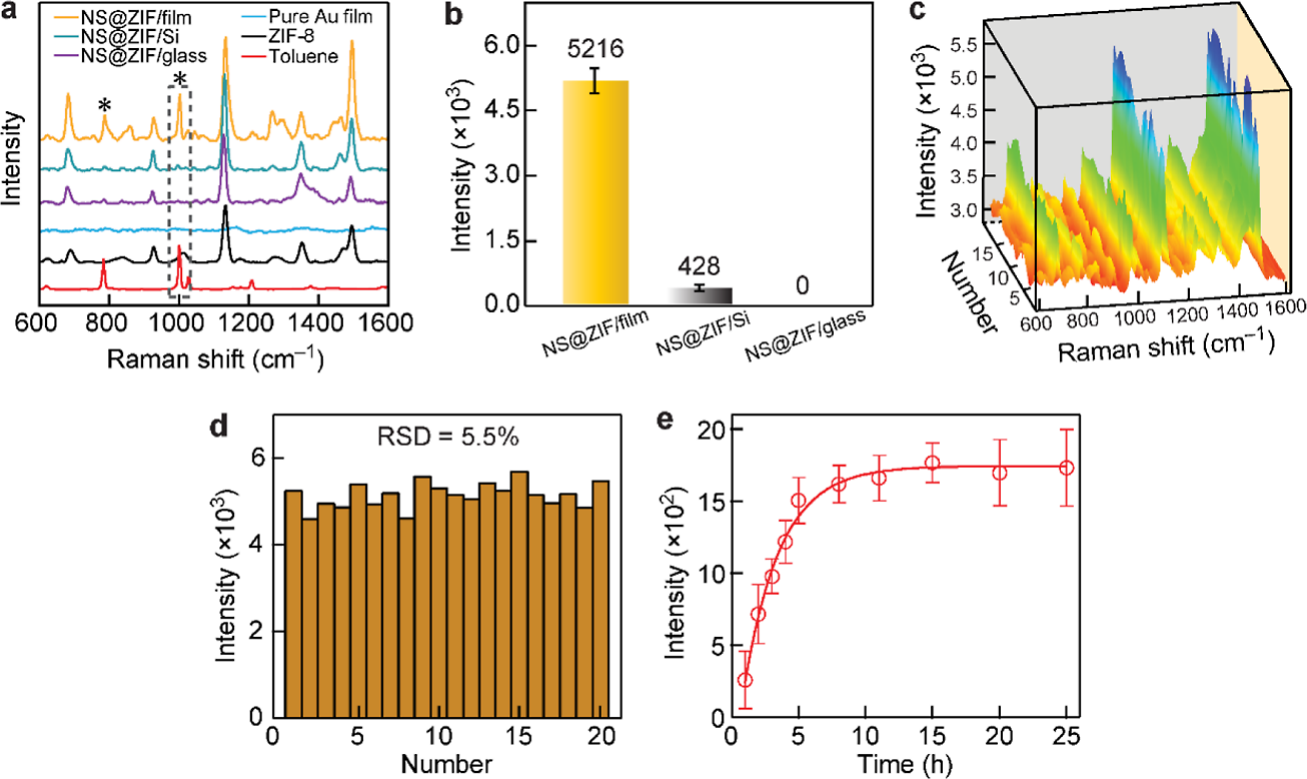
Comparison of the SERS activities. (a) SERS spectra of toluene and toluene absorbed into the different structures. (b) Integrated intensity of the (92 NS)@ZIF nanoparticles deposited on the Au film, Si, and glass substrates, respectively. The 1000 cm^–1^ peaks within the dashed box in (a) were analyzed. The ZIF thickness is 9.8 ± 2.3 nm, and the concentration of toluene is 5.6 × 1 × 10^4^ mg m^–3^. (c) Repeatability of the (92 NS)@ZIF/film structure in the detection of toluene. (d) Intensity variation of the 1000 cm^–1^ peak in (c). The average SERS intensity and relative standard deviation (RSD) of 20 random measurements are 5215 counts and 5.5%, respectively. (e) Time-dependent SERS intensity of toluene. Each data point was collected from a separate SERS substrate prepared following the same procedure. For all cases, the gap distance of the (92 NS)@ZIF/film structures is 10.2 ± 2.1 nm, and the concentration of toluene is 56 mg m^–3^.

The comparison of the Au film with Si and glass substrates further demonstrates the excellent SERS performance of our plasmon-coupled structures. The Au-film-coupled (92 NS)@ZIF nanoparticles improve the SERS intensity by more than 12 folds compared to the (92 NS)@ZIF nanoparticles supported on Si (Figure 5a,b). The SERS signals of toluene failed to be collected when optical glass slides were used as the supporting substrate. The substrate with a high refractive index provides strong image charges and leads to an intense plasmonic interaction between the original dipole of the Au NS and the image dipole in the substrate.^35,47^ The image charges in the Au film are much stronger than that in Si (*n* = 3.45) and the glass substrate (*n* = 1.52). As a result, the Au substrate of the NS@ZIF/film structure presents the best SERS performance, highlighting the superiority of the plasmon coupling in SERS detection. We further compared the SERS performances of other related structures, such as (Au NS core)@(mesoporous SiO_2_ shell) nanoparticles, sole ZIF, and bare Au NSs deposited on the Au films, respectively (Figure S29). Our plasmon-coupled (92 NS)@ZIF/film structures outperform all the other substrates, emphasizing the necessity of both the analyte-absorbing properties and the electric field enhancement in our strategy.

In general, the limitations of colloid Au nanoparticles are instability and aggregation, which would lead to poor reproducibility in quantitative SERS detection.^20,48^ The ZIF shell greatly improves the stability of the Au NSs in both water and methanol (or ethanol). The prepared NS@ZIF nanoparticles can be uniformly deposited on supporting substrates (Figures 4b and S30). As a result, the plasmon-coupled structures exhibit superior reproducibility for the SERS measurements (Figure 5c) and a small relative standard deviation in the intensity (Figure 5d). Moreover, time-dependent SERS detection was implemented to study the absorption kinetics of the vapor molecules trapped in the plasmonic nanogap hotspots (Figure 5e). For our NS@ZIF/film structures, the SERS intensity increases rapidly in the beginning and then reaches a plateau after the dynamic equilibrium of absorption and desorption is established at 5 h. It is important to point out that the evaporation process of liquid toluene occurs prior to the vapor diffusion step with the container covered during the entire measurements. Taken together, these results reveal the excellent capability of our plasmon-coupled structures in the detection of vapor molecules.

**Figure 6:**
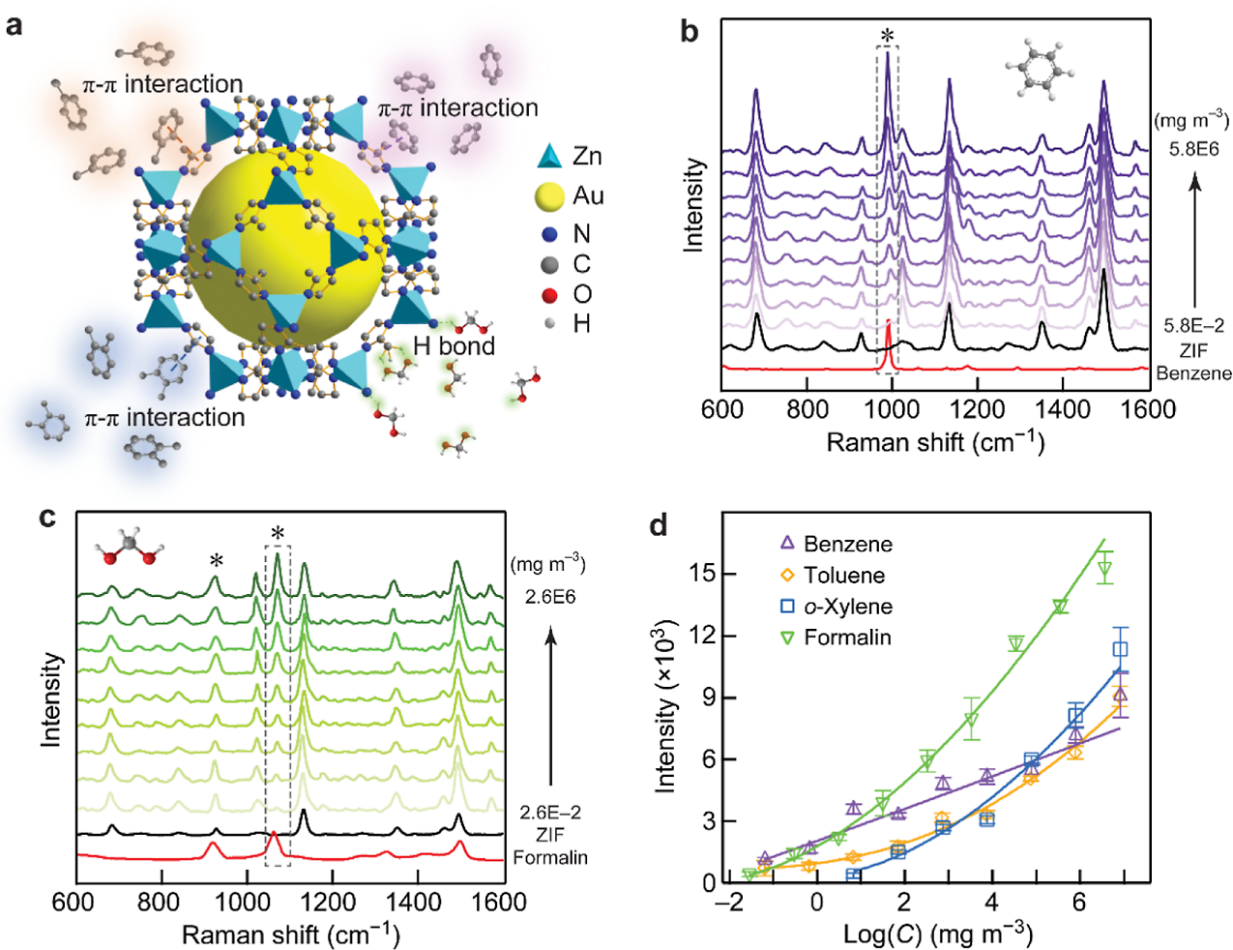
Quantitative SERS detection of VOCs. (a) Schematic illustration of the absorption principle of ZIF for VOCs. (b,c) SERS spectra of benzene (b) and formalin (c) at different concentrations (mg m^–3^) using the (92 NS)@ZIF/film structures with a gap distance of 9.7 nm. The insets show the molecular structures of benzene and formalin. The red lines are the Raman spectra of liquid benzene and formalin, and the black lines refer to the substrate signals originating from the ZIF shell. (d) Concentration-dependent SERS intensities. The characteristic peaks at 1000 cm^–1^ of toluene, 993 cm^–1^ of benzene, 733 cm^–1^ of *o*-xylene, and 1071 cm^–1^ of formalin were analyzed, as indicated by the dashed boxes in their SERS spectra. The error bars were acquired from 20 SERS spectra.

We conducted a series of quantitative measurements with benzene, toluene, *o*-xylene, and formalin using our (92 NS)@ZIF/film plasmon-coupled structures as the SERS substrates. These chemically active molecules are major harmful pollutants commonly found in newly renovated apartments and houses. They generally have a poor affinity to the Au surface, which causes difficulties in SERS detection. Fortunately, the high porosity and large surface area properties enable MOFs to interact with these chemical molecules through π–π stacking, hydrogen bonding, coordination, and electrostatic interaction.^49−51^ The ZIF shell in our architecture can trap these poor-affinity analytes into the pores with the assistance of the π–π interaction between the aromatic ring of 2-methylimidazole and the benzene ring, as well as the N–H and O–H bonds (Figure 6a). Such intermolecular interactions alter the natural vibration modes of the target analytes, producing notable shifts in their characteristic Raman peaks. These shifts serve as definitive evidence of the interactions, as shown clearly in Figure S31. The SERS responses recorded from the NS@ZIF/film substrates clearly exhibit vibrational features unique to the analytes of toluene, benzene, *o*-xylene, and formalin (Figures 6b,c and S32). The characteristic peaks of these analytes can be easily distinguished from the substrate signals. The relationships between the SERS intensity and the concentration cover a concentration range of over 8 orders of magnitude (Figures 6d and S33). The superior SERS performance of formalin is attributed to its smaller dynamic molecular size, which enables a greater number of formalin molecules to be trapped within the finite internal space of the ZIF shell. The limits of detection for these analytes satisfy the indoor air quality standard requirements. The SERS enhancement factors were estimated to range from 10^5^ to 10^7^ depending on the lateral size of the plasmonic hotspot. The sensitivity of the NS@ZIF/film structures allows the variation of the toxic vapors to be monitored. In addition, the direct detection of the vapor species gets rid of the pretreatment steps for SERS-active substrates and surpasses most SERS-based gas sensors. Last but not least, it is worth mentioning that previous studies indicate that increasing the particle density on Au films can potentially lower the detection limit of SERS.^47,52,53^ However, such systems inherently involve interparticle plasmon coupling, which is beyond the scope of this work.

## Conclusions

We have developed a facile approach for the encapsulation of individual Au NSs, NRs, and NPLs within porous ZIF shells to construct nanoparticle-on-mirror structures. The gap distance between the bottom of the Au NSs and the surface of the Au film is controlled by the ZIF thickness, which can be precisely regulated broadly. The approach relies on the appropriate control of the concentrations of CTAB and the ZIF precursors as well as the addition order of the precursors. The single-particle dark-field scattering results reveal that the out-of-plane mode of the NS@ZIF/film structures is greatly enhanced at reduced gap distances. The comparison of the SERS intensity at different excitation angles shows that the best SERS performance is achieved at an excitation angle of 60° relative to the normal of the Au film. In addition, the optimal ZIF thickness is ∼10 nm, which results from the synergistic effect of the electric field enhancement in the hotspot region and the absorption capability of the porous ZIF shell. As a SERS probe, the NS@ZIF/film substrates can detect a series of toxic VOCs at the concentration down to the 10^–2^ mg m^–3^ level. This facile and applicable strategy for trapping analytes into plasmon-coupled hotspots provides a reliable gas sensor with ultrasensitive and quantitative capability. The gas sensors are expected to be widely applicable in the biomedicine, analytical chemistry, and environmental safety fields.

## Methods

### Chemicals

HAuCl_4_·3H_2_O (99%), silver nitrate (AgNO_3_, 99%), sodium borohydride (NaBH_4_) (99%), l-(+)-ascorbic acid (AA, 99%), hydroquinone (99%), trisodium citrate, toluene (99.8%), and *o*-xylene (98%) were purchased from Sigma-Aldrich. Cetyltrimethylammonium bromide (CTAB, 99%), cetyltrimethylammonium chloride (CTAC, 97%), sodium hydroxide (NaOH, 96%), potassium iodide (KI, 99%), 2-methylimidazole (Hmim, 98%), and zinc nitrate hexahydrate (Zn(NO_3_)_2_·6H_2_O, 99%) were obtained from Aladdin. Formaldehyde solution (37%, in water, CR) was ordered from International Laboratory, USA. Methanol (99%) and benzene (AR) were purchased from RCI Labscan and Scharlab, respectively. Deionized water (H_2_O) with a resistivity of 18.2 MΩ obtained from a Direct-Q 5 UV water purification system was used in all of the experiments.

### Synthesis of the Au NSs

The Au NS samples were prepared using the seed-mediated growth method.^26^ Briefly, the seed solution was first prepared by adding a freshly prepared ice-cold NaBH_4_ solution (0.6 mL, 10 mM) into a mixture solution of HAuCl_4_ (0.25 mL, 10 mM) and CTAB (7.5 mL, 0.1 M). The resultant solution was kept under gentle stirring for 3 h at room temperature. The growth solution was prepared by the sequential addition of CTAB (2 mL, 0.1 M), HAuCl_4_ (0.8 mL, 10 mM), and AA (3 mL, 0.1 M) into H_2_O (38 mL). The seed solution (400 μL) was then added to the growth solution for the preparation of small Au NSs. The mixture was agitated by gentle inversion for 10 s and left undisturbed overnight at 35 °C. The prepared small Au NSs (∼20 nm in diameter) were washed by centrifugation (9000 rpm, 15 min) and redispersed into H_2_O (2 mL). The Au nanopolyhedrons were grown by adding the small Au NS solution (50 and 100 μL) to a mixture solution of CTAC (30 mL, 25 mM), AA (0.75 mL, 0.1 M), and HAuCl_4_ (1.5 mL, 10 mM). The mixture was placed in an air-bath shaker (45 °C) and kept for 3 h. The obtained Au nanopolyhedrons were centrifuged and redispersed in CTAB solution (30 mL, 0.02 M). HAuCl_4_ solution (0.2 mL, 10 mM) was then added into the obtained Au nanopolyhedron solution, followed by shaking at 45 °C for 2 h. The final Au NS samples were centrifuged and redispersed in H_2_O (10 mL).

### Synthesis of the (Au NS Core)@(ZIF Shell) Nanoparticles

The coating of ZIF shell on the surface of the Au NSs was carried out according to a previous report with slight modification.^25^ Typically, an aqueous Hmim solution (0.5 mL, 0.792 M) was added to a mixture solution of CTAB (72 μL, 1 mM) and the Au NSs (0.5 mL, the extinction intensity at the plasmon peak adjusted to 5.0 in a quartz cuvette of 0.2 cm optical path length), followed by shaking for 5 min. The aqueous solution of Zn(NO_3_)_2_ (0.5 mL, 14.4 mM) was then added into the reaction solution. After the mixture was shaken for 5 min, the reaction solution was left undisturbed at room temperature for 10 min. The resultant NS@ZIF nanoparticles were washed twice with methanol by centrifugation for 5 min at 3500 rpm. The NR@ZIF and NPL@ZIF nanoparticles were prepared by using the same synthetic conditions, except for the replacement of the Au NSs with the Au NRs and NPLs.

### Single-Particle Dark-Field Scattering Measurements

The surface area coverage of the NS@ZIF nanoparticles on the Au film was intentionally kept low (∼4 nanoparticles per 100 μm^2^) to facilitate the single-particle scattering measurements. Specifically, the optical density at the strongest plasmon peak of the NS@ZIF solution was standardized to be 0.5, as measured by an ultraviolet/visible/near-infrared spectrophotometer. Following this calibration, 3 μL of the sample solution was dropped onto the Au film. After being kept for 10 s, the sample-treated Au film was purged by N_2_ gas to remove the residual nanoparticles. The single-particle dark-field scattering spectra and images were recorded on an upright Olympus BX60 optical microscope, which was attached with a quartz tungsten halogen lamp (100 W), an Acton SpectraPro 2360i monochromator, and a charge-coupled device camera (Princeton Instruments Pixis 400). The camera was thermoelectrically cooled to −70 °C during the measurements. A dark-field objective (100×, numerical aperture 0.9) was used for both exciting the nanoparticles with the unpolarized white light and collecting the scattered light. The scattering spectra from the individual nanoparticles were calibrated by subtracting the background spectra taken from the adjacent regions without any nanoparticles and then dividing the difference spectra with the precalibrated response curve of the entire optical system. The scattering images were acquired by equipping a Canon EOS 600D digital camera with the eyepiece of the optical microscope. The exposure time was set at 10 s.

### FDTD Simulations

The FDTD simulations of the Au NS@ZIF nanoparticles were performed using FDTD Solution 8.7 (Lumerical Solutions). During the simulations, an electromagnetic plane wave was launched into a box containing a target nanoparticle. A mesh size of 0.5 nm was employed in calculating the extinction spectra of the NS@ZIF nanoparticles. The refractive index of the surrounding medium was set as 1.33 for water and 1.0 for air. The dielectric function of Au was obtained by fitting the measured data of Johnson and Christy. The refractive index of the ZIF shell was adjusted to match our measured extinction spectra and set to be 1.42. The sizes of the Au NS and ZIF shells were set according to the diameter and thickness obtained from the TEM images. The propagation direction of the excitation light was set perpendicular to the Au film in the simulation of the extinction and single-particle dark-field scattering spectra and changed relative to the normal of the Au film in the angle-varying scattering simulations.

### SERS Measurements of VOCs

The SERS substrates were prepared in advance. The extinction intensities of the strongest plasmon peaks for the Au NSs and the NS@ZIF nanoparticles were adjusted to 5.0 in quartz cuvettes with 0.2 cm optical path length. The Au film, Si, and glass substrates of 6 × 6 mm^2^ were slightly adhered onto glass slides (6 × 30 mm^2^, Ted Pella). The Au NSs (12 μL, in water), ZIF (6 μL, 20 mg mL^–1^, in methanol), and NS@ZIF nanoparticles (12 μL, in methanol) were separately drop-cast onto the substrates. These samples were dried in a vacuum. The resultant substrates were placed in sealed vials (3 mL) with liquid analytes (20 μL for each) added in advance. The vials were sealed with lids and parafilm. The sealed vials were then incubated in an oven at 60 °C for 6 h. The substrates with the analytes were finally measured by using a portable Raman spectrometer.

## Supporting Information Available

Characterization, synthesis of the Au NRs and NPLs, discussion of the SERS results, TEM images, thicknesses of the ZIF coatings, extinction spectra, TEM elemental mapping and analysis results, scattering spectra and images, electric field enhancement results, SERS spectra and results, SEM images, and plasmon wavelengths (PDF)
