# Supplementary material for: Metal–Organic Framework-Enabled Trapping of Volatile Organic Compounds into Plasmonic Nanogaps for Surface-Enhanced Raman Scattering Detection
Source: ACS Nano. 2024 Apr 17;18(17):11234–44. doi: 10.1021/acsnano.4c00208 (PMC11064218; doi:10.1021/acsnano.4c00208)
Supplement: Supplementary file 1 — nn4c00208_si_001.pdf [file nn4c00208_si_001.pdf]

Supporting Information

Metal–Organic Framework-Enabled Trapping of Volatile Organic Compounds into Plasmonic Nanogaps for Surface-Enhanced Raman Scattering Detection

Yi Liu,[1] Ka Kit Chui,[1] Yini Fang,[1] Shizheng Wen,[2] Xiaolu Zhuo,[3] and Jianfang Wang[1,]*

[1]Department of Physics, The Chinese University of Hong Kong, Shatin, Hong Kong SAR 999077, China

[2]Jiangsu Province Key Laboratory of Modern Measurement Technology and Intelligent Systems, School of Physics and Electronic Electrical Engineering, Huaiyin Normal University, Huaian 223300, China

[3]School of Science and Engineering, The Chinese University of Hong Kong (Shenzhen), Shenzhen 518172, China

*Email: jfwang@phy.cuhk.edu.hk

METHODS

Characterization. Transmission electron microscopy (TEM) images were taken on an FEI Tecnai G2 Spirit Twin microscope operated at 120 kV. Extinction spectra were measured on a Lambda 950 ultraviolet/visible/near-infrared spectrophotometer. Scanning electron microscopy (SEM) images were taken on an FEI Quanta 400 FEG microscope operated at 40 kV. High-angle annular dark-field scanning transmission electron microscopy (HAADF-STEM) imaging and elemental mapping were carried out on an FEI Tecnai F20 (G2 F20 S-TWIN FA) microscope operated at 200 kV and equipped with an Oxford energy-dispersive X-ray (EDX) analysis system. X-ray diffraction (XRD) measurements were performed on a SmartLab 9 kW diffractometer equipped with Cu k_α radiation. The Au films were fabricated by first depositing a 5 nm Ti adhesion layer and then a 100 nm Au layer onto smooth Si substrates by electron-beam evaporation (EBS-500, Junsun Tech Co., Taiwan). Single-particle dark-field scattering spectra and images were recorded on an upright optical microscope (Olympus, BX60) that was integrated with a quartz-tungsten-halogen lamp (100 W), a monochromator (Acton, SpectraPro 2360i), and a charge-coupled device camera (Princeton Instruments, Pixis 400, cooled to −70 °C). In the measurements, a dark-field objective (100×, numerical aperture 0.9) was employed for both exciting the individual NS@ZIF nanoparticles on the Au film with the unpolarized white light and collecting the scattered light. The scattering spectra of the Au-film-supported NS@ZIF nanoparticles were corrected by first subtracting the background spectrum taken from the adjacent region without any nanoparticles and then dividing it with the pre-calibrated response curve of the entire optical system. The exposure time was set at 10 s. The SERS spectra were measured using a portable Raman spectrometer (Ocean Optics SR-510 Pro) at an excitation wavelength of 785 nm, a laser power of 105 mW, and an acquisition time of 5 s.

Synthesis of the Au NRs. The Au NRs were grown according to a previously reported work.[1] A freshly prepared, ice-cold $NaBH_4$ aqueous solution (0.6 mL, 10 mM) was added into a mixture solution of $HAuCl_4$ (0.25 mL, 10 mM) and CTAB (9.75 mL, 0.1 M) to prepare the seed solution. For the preparation of the growth solution, $HAuCl_4$ solution (2 mL, 10 mM) and $AgNO_3$ solution (0.6 mL, 10 mM) were sequentially added into CTAB solution (38 mL, 0.1 M). The obtained growth mixture was shaken for 30 s. The reducing agent, an aqueous hydroquinone solution (2 mL, 0.1 M), was then added into the growth solution, followed by gently shaking until it became colorless. The pre-prepared seed solution (2 mL) was quickly added into the

above growth solution. After being vigorously shaken for 10 s, the growth solution was aged at 30 °C for 6 h. The grown Au NRs were collected through centrifugation at 5000 rpm for 10 min and redispersed in H_2O (10 mL).

Synthesis of the Hexagonal Au NPLs. The hexagonal Au NPL sample was synthesized according to a reported work.[2] The seed solution was prepared by adding $HAuCl_4$ solution (1 mL, 10 mM) and trisodium citrate solution (1 mL, 10 mM) into H_2O (36 mL), followed by quick addition of a freshly prepared, ice-cold $NaBH_4$ solution (1 mL, 0.1 M). The resultant seed solution was vigorously shaken for 2 min and aged at 30 °C for 2 h. Three solutions were prepared for the growth of the Au NPLs. Solutions 1 and 2 were made by sequentially mixing CTAB (9 mL, 0.05 M), $HAuCl_4$ (0.25 mL, 10 mM), NaOH (0.05 mL, 0.1 M), KI (0.05 mL, 0.01 M), and AA (0.05 mL, 0.1 M). Solution 3 was made by enlarging solution 1 or 2 by 10 times. The growth of the Au NPLs was started by adding the seed solution (1 mL) to solution 1, followed by gently shaking for 5 s. The resultant solution 1 (1 mL) was added to solution 2, followed by shaking for 5 s. All the resultant solution 2 was then added to solution 3. The obtained mixture solution 3 was vigorously shaken and then left undisturbed at room temperature for 24 h. The supernatant was gently removed. The obtained precipitate was then redispersed into H_2O (40 mL). The overgrowth solution was prepared by mixing CTAB (0.25 mL, 0.1 M), $HAuCl_4$ (60 µL,10 mM), AA (30 µL, 0.1 M), and deionized H_2O (1.66 mL) in sequence. The precipitate solution (0.5 mL) was added to initiate the overgrowth of Au NPLs. The resultant mixture solution was placed in an oven at 30 °C and aged for 10 h. The Au NPL sample was obtained by centrifugation and redispersed into H_2O.

Discussion on the SERS Measurement Results. ZIF-8 nanocrystals show the characteristic Raman peaks at 682, 928, 1134, 1360, and 1495 cm^{-1}, which arise from the imidazolium ring puckering, C–H out-of-plane bending, C–N stretching, methyl bending, and a mixture of the vibration modes of C–N stretching and N–H wagging of 2-methylimidazole, respectively.[3,4] Benzene has a strong peak at 993 cm^{-1}, which is originated from the benzene ring breathing vibration mode.[5,6] Toluene exhibits the distinctive peaks at 783, 1000, and 1207 cm^{-1}, which correspond to C–H bending, symmetric ring stretching, and methyl stretching, respectively.[7,8] *o*-Xylene has characteristic peaks at 579, 733, 1050, and 1219 cm^{-1}, which are the out-of-plane bending mode of C–H, the out-of-plane bending mode of C–C–C in the ring, ring breathing, and a mixture mode of methyl stretching and C–H in-plane bending, respectively.[7,9] The vibrational

peaks of formalin at 924, 1064, and 1498 cm^{-1} are originated from C–OH symmetric stretching vibrations, the $-CH_2$ rocking mode, and $-CH_2$ scissoring vibrations, respectively.[10,11]

The SERS enhancement factor was calculated according to[12]

$$\mathrm{EF} = \frac{I_{\mathrm{SERS}}/N_{\mathrm{SERS}}}{I_{\mathrm{Raman}}/N_{\mathrm{Raman}}} \tag{1}$$

where I_{SERS} and I_{Raman} are the peak intensities in the SERS and normal Raman measurements, respectively, N_{SERS} and N_{Raman} are the numbers of the molecules contributing to the Raman signal in the SERS measurement using the Au-film-supported NS@ZIF nanoparticles and the normal Raman measurement, respectively. Take toluene and its 1000 cm^{-1} peak as an example. An ethanolic solution of toluene (20 µL, 9.4×10^{-7} M) was added into the vial with the (NS@ZIF)-on-film structure inside for the SERS measurements. In the sealed vial, the (NS@ZIF)-on-film structure reached an adsorption–desorption equilibrium. The SERS substrate generated a SERS signal of 701 counts. Only a fraction of the analyte molecules was absorbed by the ZIF shell. The thickness of the ZIF shell is 10 nm. We assume that the hotspot has a lateral size of d (nm) in diameter. The amount of absorbed toluene is assumed to be 100 mg per g of ZIF according to a previous study.[13] The density of ZIF is 0.94 g cm^{-3}, and the molecular weight of toluene is 92 g mol^{-1}. Therefore, the number of toluene molecules absorbed into each plasmonic nanogap hotspot can be estimated according to

$$\begin{aligned} N_{\mathrm{spot}} &= \frac{\pi\frac{d^2}{4}\times 10\ \mathrm{nm}\times 0.94\ \mathrm{g\ cm^{-3}}\times 0.1\ \mathrm{g}}{92\ \mathrm{g\ mol^{-1}}} \times 6.02\times 10^{23} \\ &= 4.83d^2\ (d\!:\mathrm{nm}) \end{aligned} \tag{2}$$

The number density of the core@shell nanoparticles is 20 per µm^2. The laser spot area on the SERS substrate for our portable Raman spectrometer is 3.14 mm^2. The number of the probed toluene molecules on the SERS substrate can then be estimated as

$$\begin{aligned} N_{\mathrm{SERS}} &= 4.83d^2 \times 20 \times 3.14 \times 10^6 \\ &= 3.03 \times 10^8 d^2\ (d\!:\mathrm{nm}) \end{aligned} \tag{3}$$

A regular Raman signal of 6763 counts was obtained by the direct measurement of pure toluene (5 mL, 9.4 M) in a glass vial. The probe head of the portable Raman spectrometer has a numerical aperture of 0.22. Its depth of focus was assumed to be 5 µm. The number of the probed toluene molecules in the regular measurement is therefore

$$\begin{aligned} N_{\mathrm{Raman}} &= 3.14\ \mathrm{mm^2} \times 5\ \mathrm{\mu m} \times 9.4\ \mathrm{mol\ L^{-1}} \times 6.02 \times 10^{23} \\ &= 8.88 \times 10^{16} \end{aligned} \tag{4}$$

As a result, the enhancement factor of our plasmon-coupled structure can be calculated as follows

$$\mathrm{EF} = \frac{701/(3.03\times10^{8}\times d^{2})}{6763/(8.88\times10^{16}\ \mathrm{molecules})}$$

$$= \frac{3.04\times10^{7}}{d^{2}}\ (d\colon \mathrm{nm}) \quad (5)$$

The estimated enhancement factor is highly dependent on the lateral size of the hotspot. If d is assumed to be 1 nm, 5 nm, and 10 nm, the enhancement factors will be 3.0×10^7, 1.2×10^6, and 3.0×10^5, respectively.

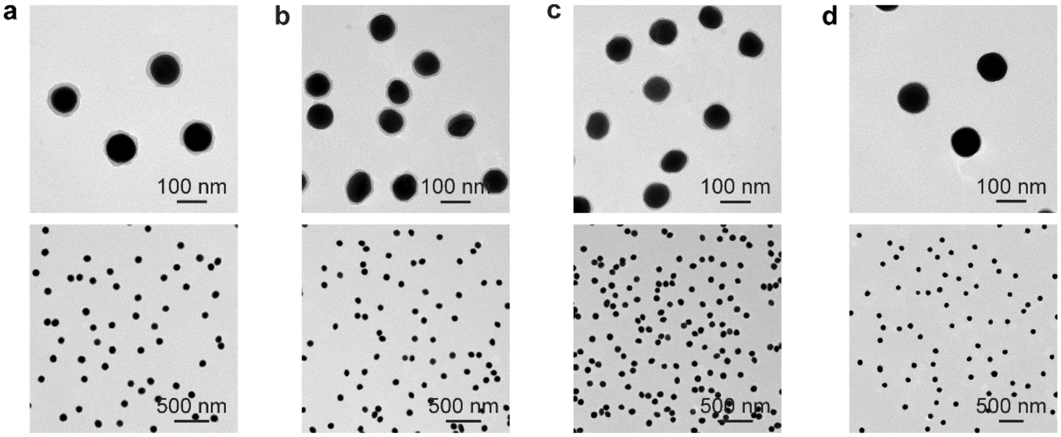

Figure S1. TEM images of the (92 NS)@ZIF nanoparticles. (a–d) Nanoparticles prepared at the CTAB (1 mM) volumes of 0 (a), 72 (b), 144 (c), and 288 µL (d). The corresponding ZIF thicknesses are 10.6 ± 2.6, 7.2 ± 2.0, 4.9 ± 1.5, and 3.5 ± 0.9 nm, respectively. The concentrations of added Hmim and Zn^{2+} were 0.792 M (0.5 mL) and 14.4 mM (0.5 mL). The Hmim solution was added before the Zn^{2+} solution. The reaction time was 15 min. Upper: high-magnification TEM images. Lower: low-magnification TEM images.

The prepared Au NSs and NS@ZIF nanoparticles possess uniform morphologies and narrow size distributions. The starting Au NS sample was dispersed in water after centrifugation. The residual concentration of CTAB in the Au NS solution was estimated to be less than 10^{-7} M,[14] which can be neglected compared with the additionally added CTAB. Without the supply of additional CTAB, the CTAB concentration in the reaction mixture was below its critical micelle concentration (9×10^{-4} M). The low CTAB concentration led to a small packing density of CTAB bilayer on the surface of the Au NSs. The precursors of ZIF-8 could be easily attached to

the Au NS surface. ZIF nanocrystals thereafter nucleated and grew on the surface of the metal nanoparticle because of a favorable interaction between the metal surface and the ZIF nanocrystals.[15] An increase in the CTAB concentration enhanced the packing density of the CTAB bilayer adsorbed on the surface of the Au NSs. The deposition of the ZIF precursors was therefore hindered. At the same time, CTAB molecules with the long hydrocarbon chains were adsorbed preferentially on the {100} facets of the ZIF nanocrystals to minimize the surface energy and suppress the ZIF crystallization.[15,16] The increase in the CTAB concentration also promoted the formation of CTAB micelles in the solution. The free CTAB micelles in the solution competed with the CTAB layers on the metal surface and caused the self-nucleation and growth of ZIF nanocrystals. However, these self-nucleated ZIF nanocrystals were small and could be easily removed by centrifugation. As a result, under these situations, the higher the CTAB concentration, the thinner the ZIF shell. Further increase of the CTAB concentration would severely inhibit the growth of ZIF on the Au NS surface. Therefore, the successful synthesis of the NS@ZIF nanoparticles requires careful adjustment of the CTAB amount around the critical micelle concentration.

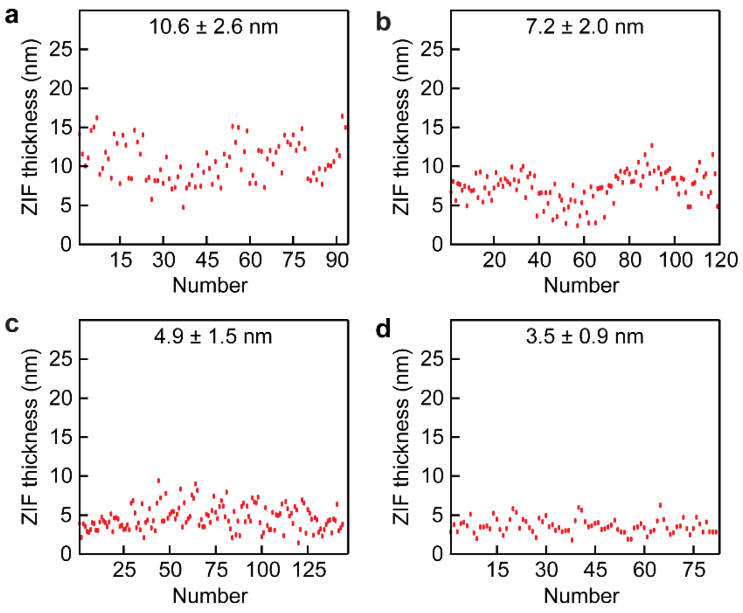

Figure S2. ZIF thickness distributions. (a–d) Corresponding to the four (92 NS)@ZIF nanoparticle samples shown in Figure S1.

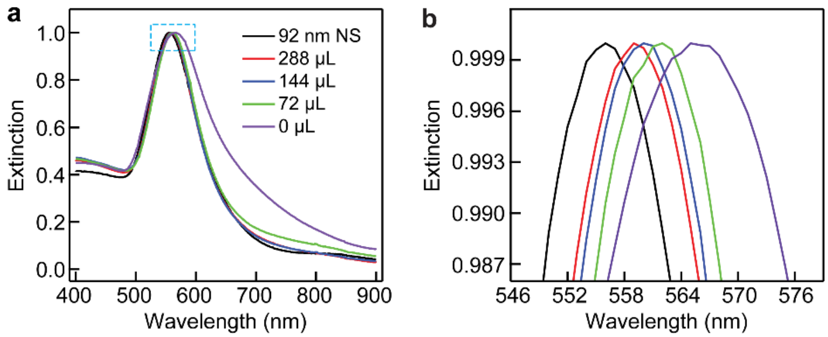

Figure S3. Normalized extinction spectra of the (92 NS)@ZIF nanoparticles. (a) Extinction spectra. (b) Zoomed-in view of the extinction peaks. The four (92 NS)@ZIF nanoparticle samples are those shown in Figure S1.

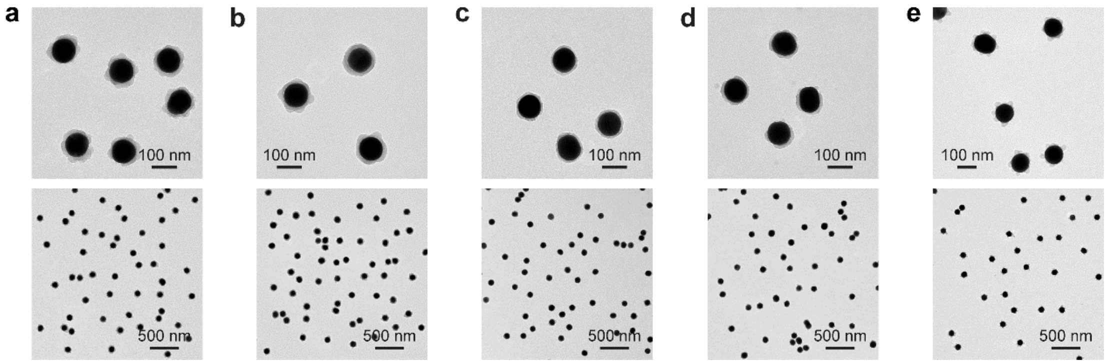

Figure S4. (92 NS)@ZIF nanoparticles. (a–e) Nanoparticles synthesized by the addition of Hmim/Zn^{2+} at the concentrations of 1.056 M/19.2 mM (a), 0.924 M/16.8 mM (b), 0.792 M/14.4 mM (c), 0.66 M/12 mM (d), and 0.528 M/9.6 mM (e). The corresponding ZIF thicknesses are 12.5 ± 3.5, 10.8 ± 2.6, 7.8 ± 2.0, 9.0 ± 2.5, and 11.8 ± 5.7 nm, respectively. The Hmim solution was added before the Zn^{2+} solution. The volumes of the Hmim and Zn^{2+} solutions were both 0.5 mL. The added CTAB solution was 72 µL (1 mM), and the reaction time was 15 min.

The concentrations of the ZIF precursors play an important role in the control of the shell thickness. A high precursor concentration promoted the nucleation and growth rates of ZIF nanocrystals and resulted in a thicker ZIF shell. At low precursor concentrations, the nucleation and growth of ZIF were restricted under the action of CTAB. Small ZIF nanocrystals were formed and randomly deposited on the surface of the Au NSs, which led to a large distribution in the shell thickness. The results indicate that the concentrations of CTAB and the ZIF precursors are two crucial factors for the coating of ZIF on the surface of the Au NSs.

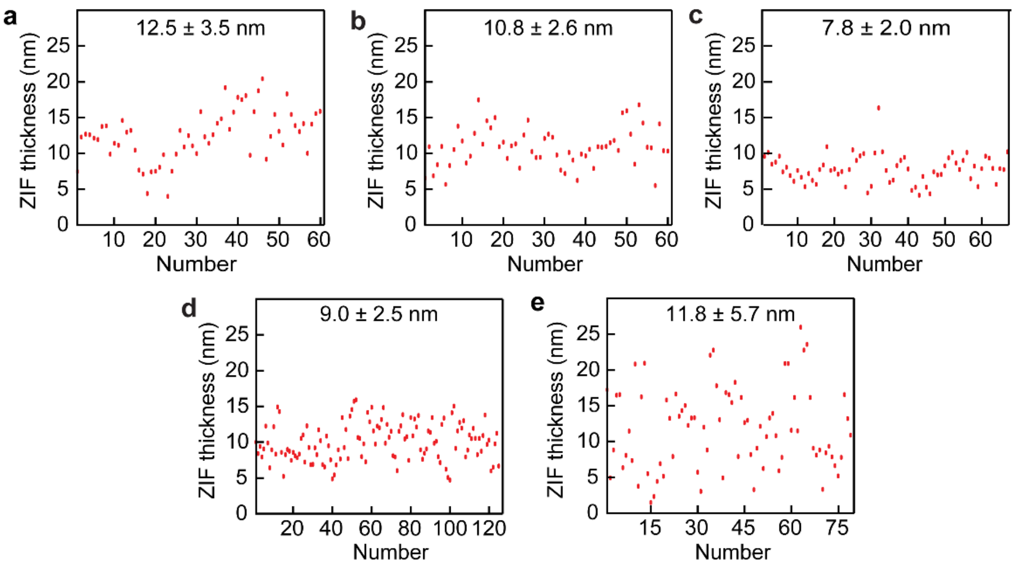

Figure S5. ZIF thickness distributions. (a–e) Corresponding to the five (92 NS)@ZIF nanoparticle samples shown in Figure S4.

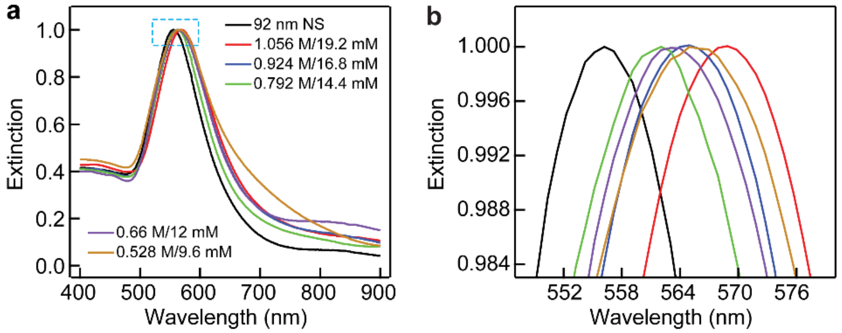

Figure S6. Normalized extinction spectra of the (92 NS)@ZIF nanoparticles. (a) Extinction spectra. (b) Zoomed-in view of the extinction peaks. The five (92 NS)@ZIF nanoparticle samples are those shown in Figure S4.

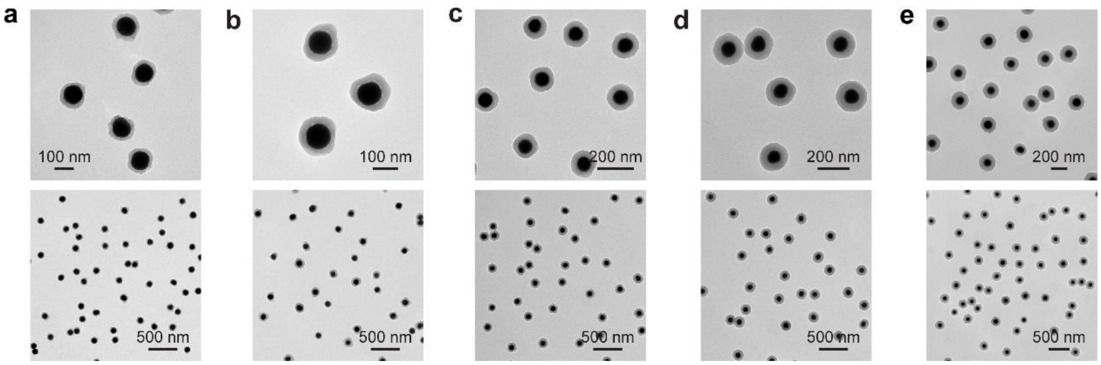

Figure S7. (92 NS)@ZIF nanoparticles. (a–e) Nanoparticles prepared by adding the Zn^{2+} solution (9.6 mM, 0.5 mL) before the Hmim solution (0.528 M, 0.5 mL) and adding CTAB solution (1 mM) at 200 (a), 150 (b), 100 (c), 50 (d), and 0 μL (e). The ZIF thicknesses are 17.6 ± 5.4, 25.4 ± 7.7, 34.8 ± 4.5, 45.2 ± 4.0 nm, and 59.5 ± 4.9, respectively. The reaction time was 15 min.

The CTAB-stabilized Au NSs attract Hmim and Zn^{2+} differently. Hmim molecules are weakly negatively charged because of the hydrolysis and the lone-pair electrons of the nitrogen atoms. The CTAB-capped Au NSs are positively charged even at a low packing density of the CTAB bilayer. The positively charged NSs attract Hmim molecules, which are distributed uniformly around the surface of the Au NSs. At the same time, numerous Hmim molecules are freely distributed in the solution because of the high concentration of Hmim. When a small amount of Zn^{2+} solution was added into the reaction solution, most Zn^{2+} ions were consumed by the free Hmim molecules dissolved in the solution. A small number of Zn^{2+} ions approached the surface of the Au NSs and bonded with the Hmim molecules to form ZIF nanocrystals. The slow nucleation and growth rates of ZIF nanocrystals resulted in a small shell thickness. In contrast, when Zn^{2+} solution was first added into the Au NS solution, the Zn^{2+} ions can disrupt the CTAB bilayer,[17] enabling the Zn^{2+} ions to adsorb directly on the exposed surface of the Au NSs. The amount of the Zn^{2+} ions distributed freely in the solution will be small. After the addition of the Hmim solution at a concentration higher than Zn^{2+}, the small diffusion barrier of Hmim in the solution enabled a large fraction of Hmim molecules to reach the Au NS surface and bond with the Zn^{2+} ions adsorbed on the Au NSs. The enhanced growth rate therefore yielded a thicker shell around the Au NSs. In this case, the shell thickness can also be adjusted through the

interaction between the hydrocarbon chain of CTAB with the {100} facets of the ZIF nanocrystals.

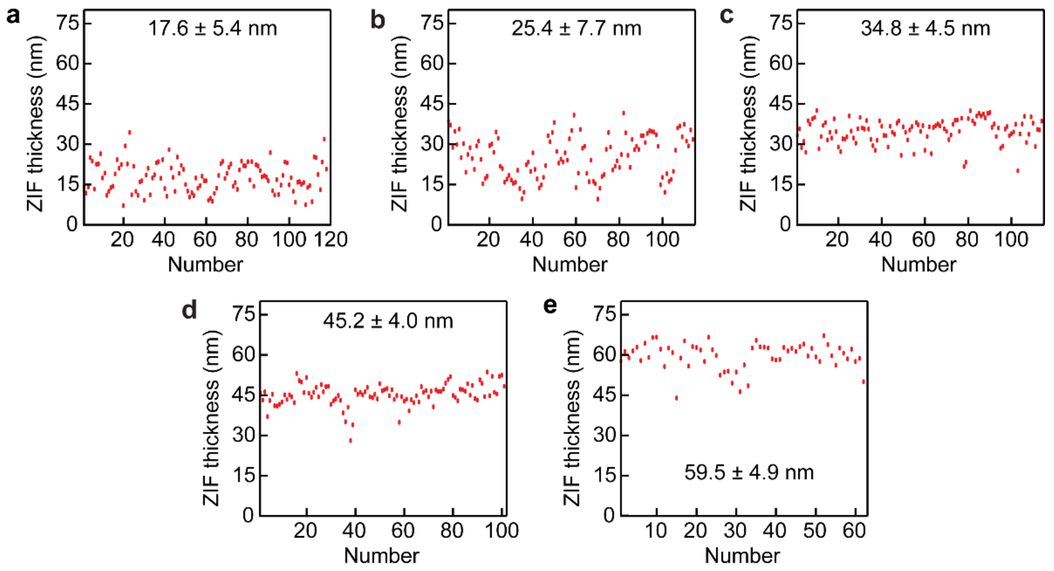

Figure S8. ZIF thickness distributions. (a–e) Corresponding to the five (92 NS)@ZIF nanoparticle samples shown in Figure S7.

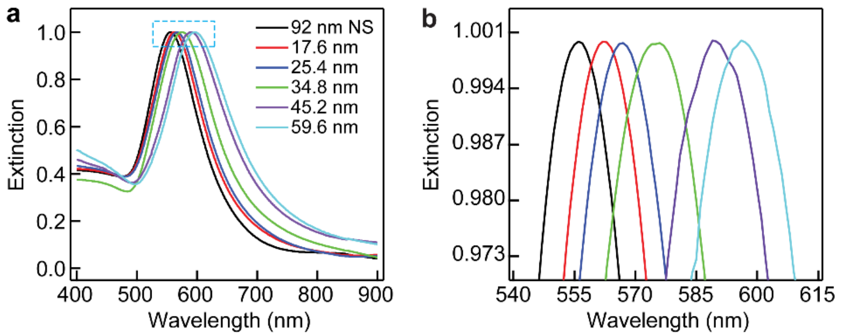

Figure S9. Normalized extinction spectra of the (92 NS)@ZIF nanoparticles. (a) Extinction spectra. (b) Zoomed-in view of the extinction peaks. The five (92 NS)@ZIF nanoparticle samples are those shown in Figure S7.

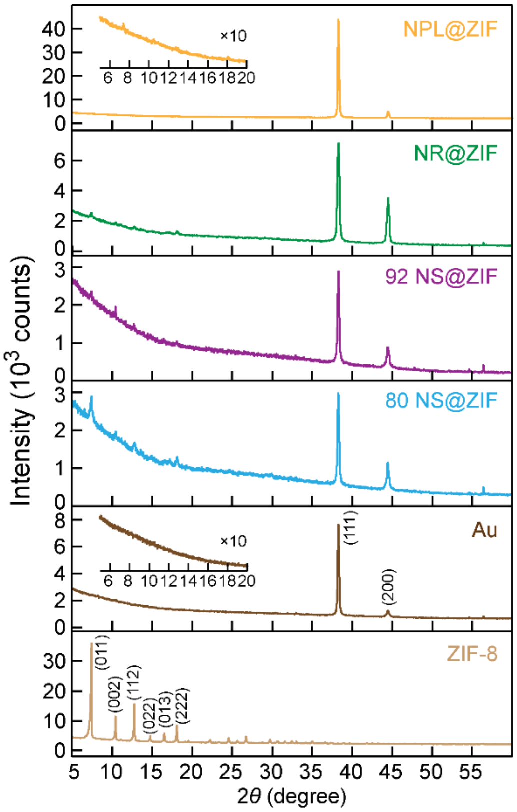

Figure S10. XRD patterns of the Au NSs, ZIF-8, and (Au core)@(ZIF shell) nanoparticles.

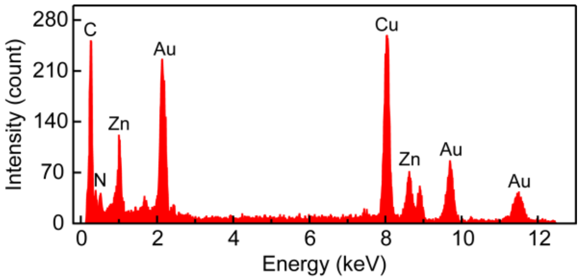

Figure S11. Energy-dispersive X-ray analysis of the (92 NS)@ZIF-9.6 nanoparticles. The Au signal comes from the Au NSs. The C, N, and Zn signals are derived from the ZIF-8 shell. In addition, the Cu signal is caused by the Cu TEM grid used in the experiments.

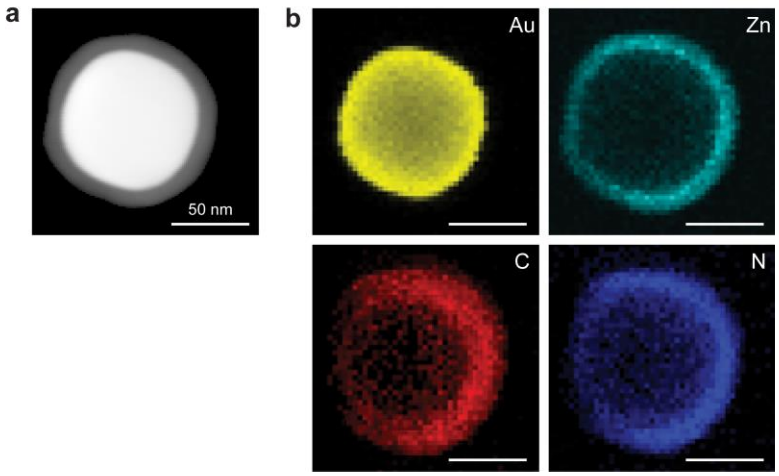

Figure S12. Characterization of the (92 NS)@ZIF-9.6 nanoparticles. (a) High-angle annular dark-field scanning transmission electron microscopy image. (b) Elemental mapping images for the Au, Zn, C, and N elements.

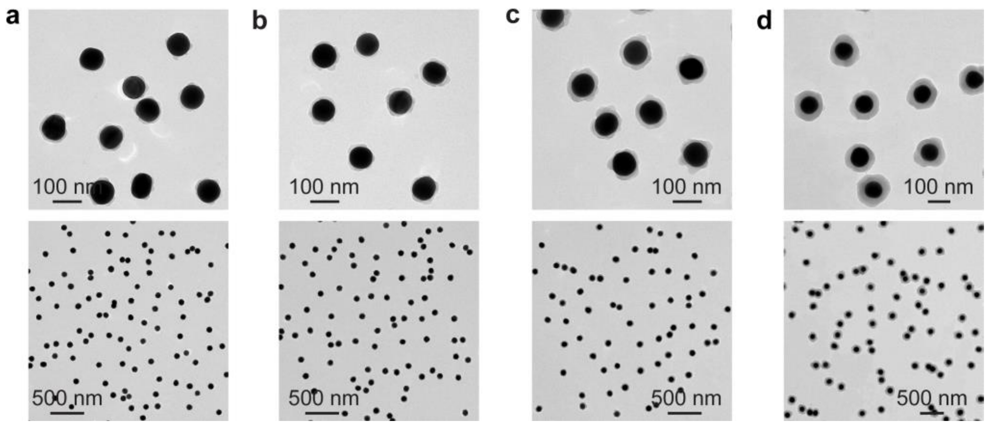

Figure S13. TEM images of the (80 NS)@ZIF nanoparticles with different shell thicknesses. (a) 4.8 ± 1.7 nm. (b) 8.3 ± 2.1 nm. (c) 12.8 ± 3.0 nm. (d) 25.1 ± 5.7 nm. Top row: high-magnification images. Bottom row: low-magnification images. The average diameter of the Au NSs is 80.2 ± 3.0 nm.

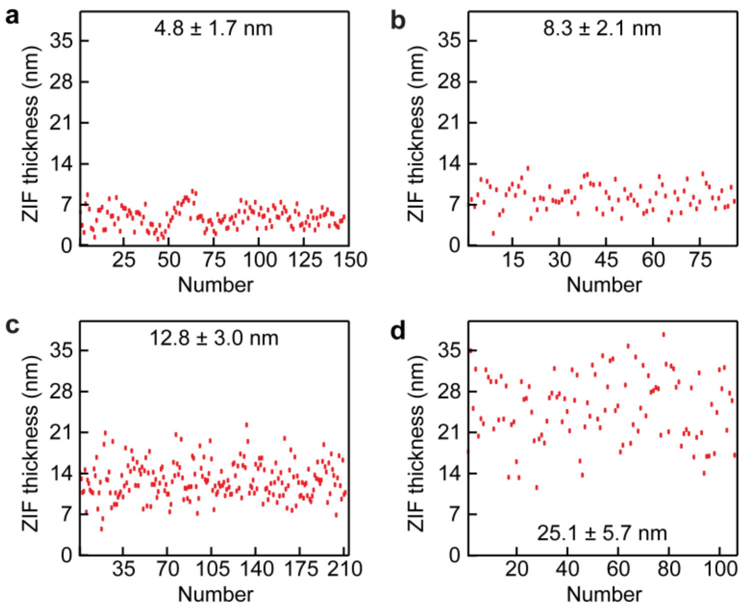

Figure S14. ZIF thickness distributions of the (80 NS)@ZIF nanoparticles.

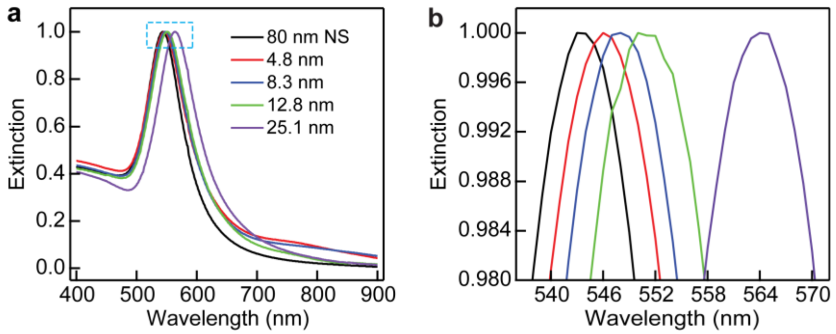

Figure S15. Normalized extinction spectra of the (80 NS)@ZIF nanoparticles with different shell thicknesses. (a) Experimental extinction spectra. (b) Zoomed-in view of the extinction peaks.

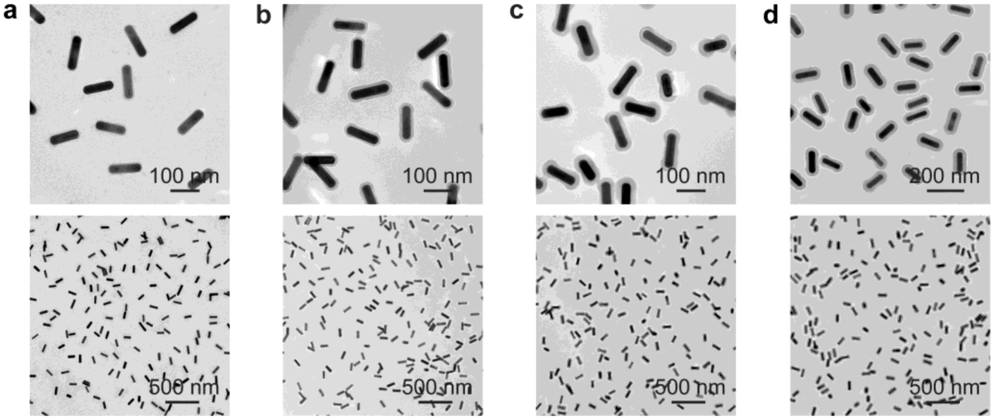

Figure S16. TEM images of the NR@ZIF nanoparticles with different shell thicknesses. (a) 3.6 ± 0.9 nm. (b) 9.5 ± 2.4 nm. (c) 15.7 ± 3.5 nm. (d) 21.0 ± 3.0 nm. Top row: high-magnification images. Bottom row: low-magnification images. The average length and width of the Au NRs are 109.6 ± 12.4 nm and 30.3 ± 2.2 nm, respectively.

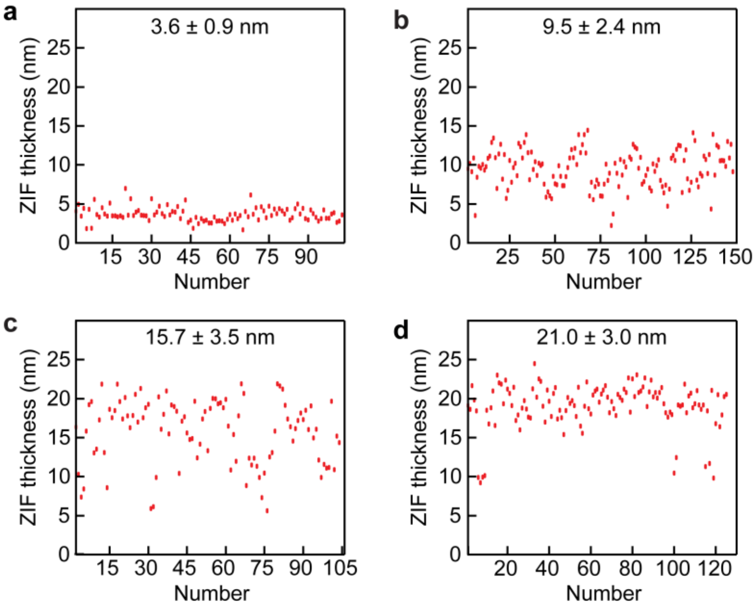

Figure S17. ZIF thickness distributions of the NR@ZIF nanoparticles.

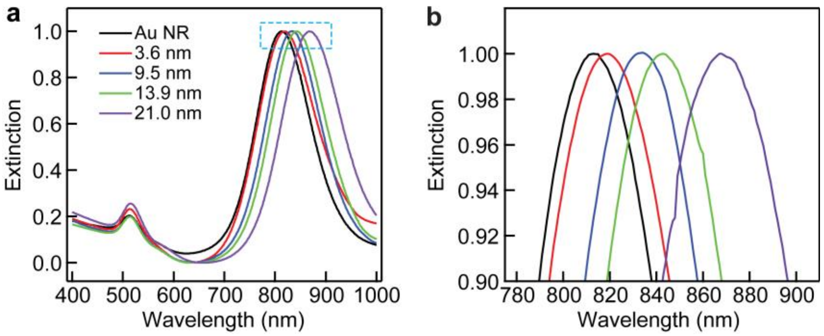

Figure S18. Normalized extinction spectra of the NR@ZIF nanoparticles with different shell thicknesses. (a) Experimental extinction spectra. (b) Zoomed-in view of the extinction peaks.

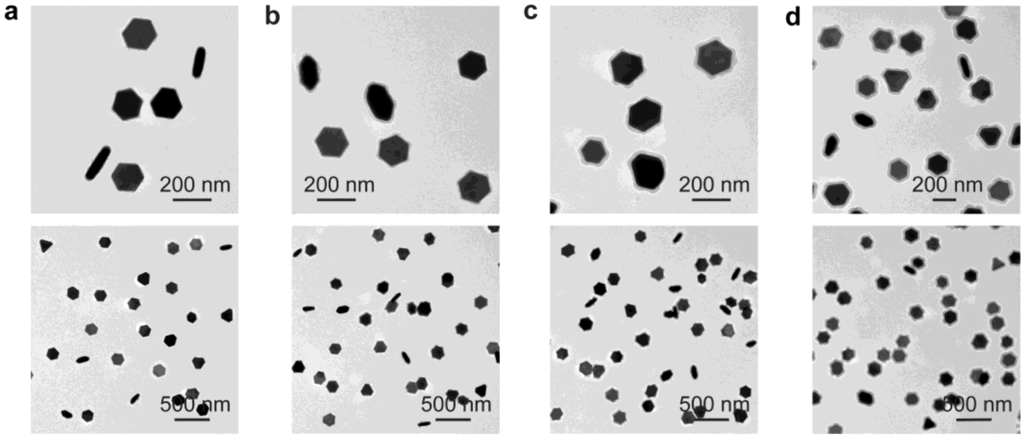

Figure S19. TEM images of the NPL@ZIF nanoparticles with different shell thicknesses. (a) 5.9 ± 2.0 nm. (b) 10.2 ± 2.3 nm. (c) 14.8 ± 3.0 nm. (d) 23.7 ± 3.3 nm. Top row: high-magnification images. Bottom row: low-magnification images. The average lateral size and thickness of the Au NPLs are 148 ± 8 nm and 52.5 ± 4.7 nm, respectively. The lateral size means the distance between two opposite parallel edges.

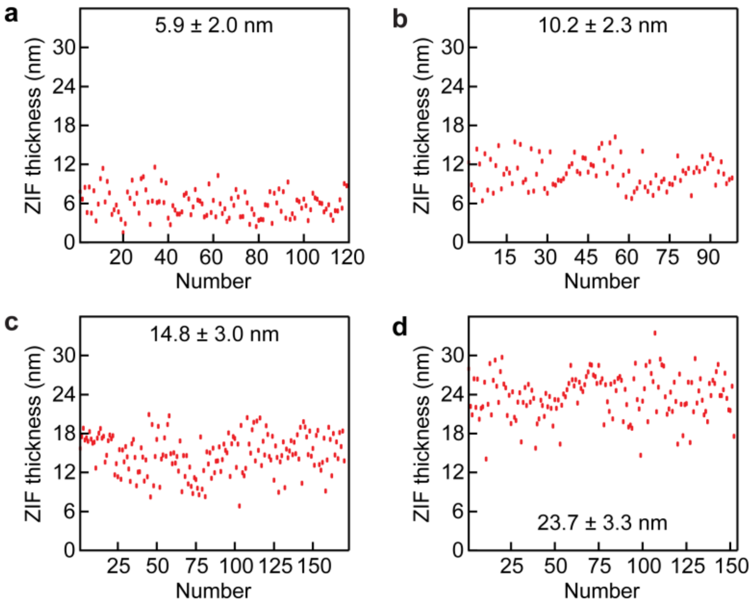

Figure S20. ZIF thickness distributions of the NPL@ZIF nanoparticles.

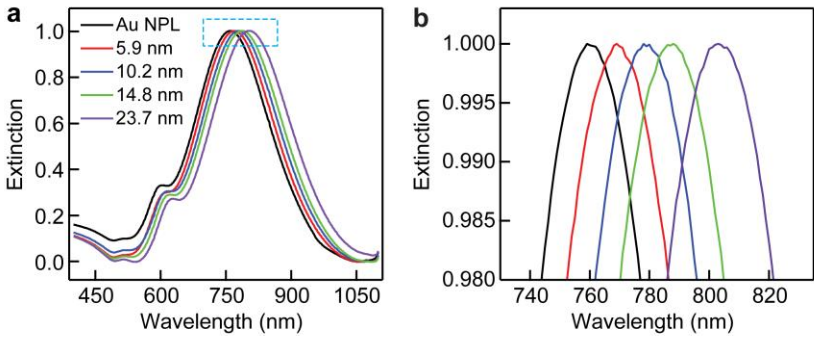

Figure S21. Normalized extinction spectra of the NPL@ZIF nanoparticles with different shell thicknesses. (a) Experimental extinction spectra. (b) Zoomed-in view of the extinction peaks.

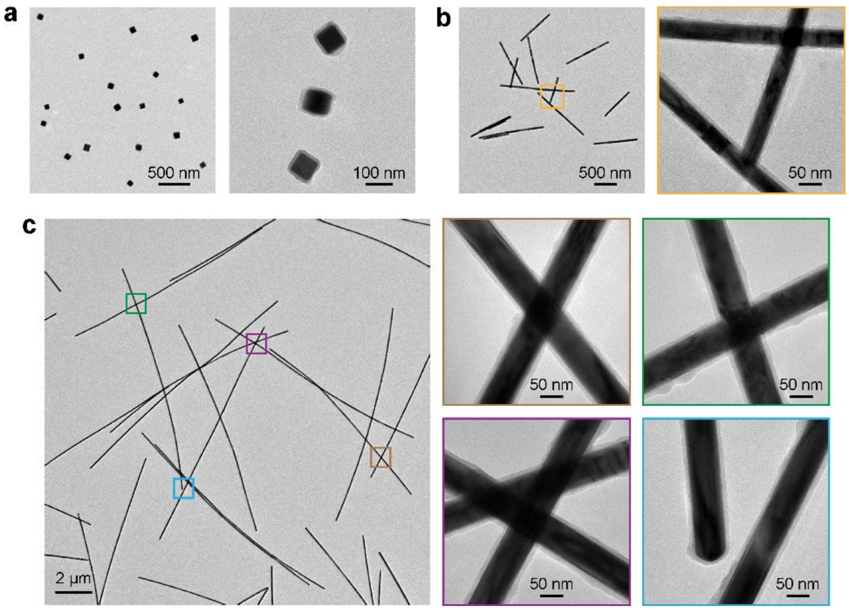

Figure S22. Coating ZIF-8 on Ag nanoparticles. (a) Low- (left) and high-magnification (right) TEM images of the (Ag nanocbue)@ZIF nanoparticles. The average edge length of the Au nanocubes is 82 nm. (b) TEM images of the (Ag nanorod)@ZIF nanoparticles. The average length of the Ag nanorods is ~1 μm. The zoomed-in TEM image was taken from the boxed region. (c) TEM images of the (Ag nanorod)@ZIF nanoparticles. The average length of the Ag nanorods is ~10 μm. The zoomed-in TEM images were taken from the boxed regions.

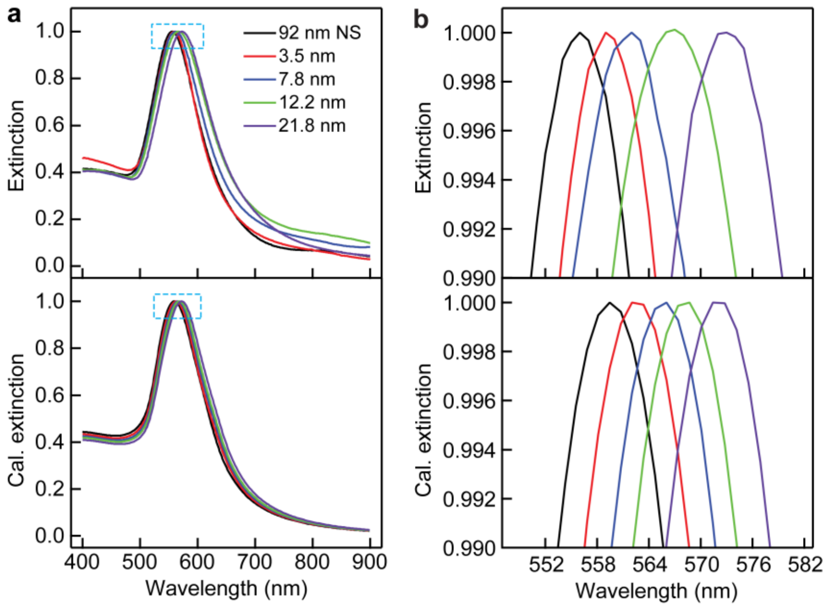

Figure S23. Normalized extinction spectra of the (92 NS)@ZIF nanoparticles with varying shell thicknesses. (a) Measured (top) and FDTD-simulated (bottom) extinction spectra. (b) Zoomed-in views of the extinction peaks in (a).

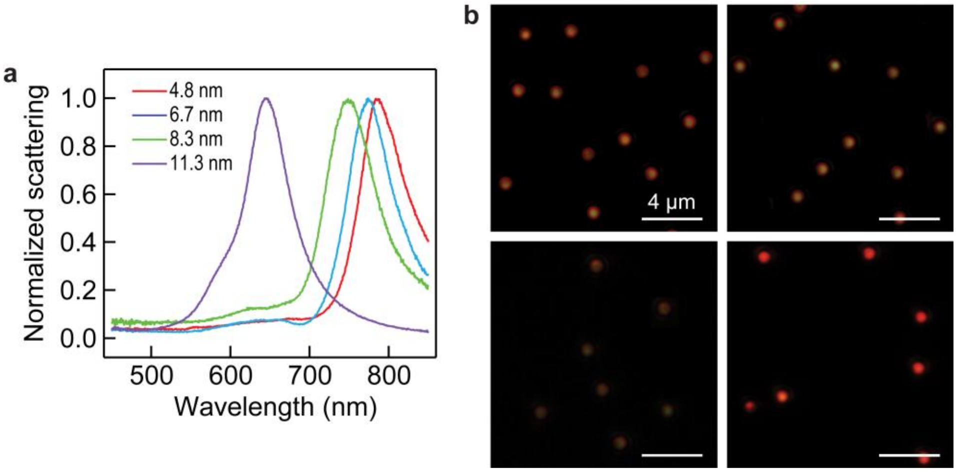

Figure S24. Plasmonic properties of the (80 NS)@ZIF/film structures with different gap distances. (a) Single-particle dark-field scattering spectra. (b) Dark-field scattering images. The gap distances are 4.8 nm (top left), 6.7 nm (top right), 8.3 nm (bottom left), and 11.3 nm (bottom right), respectively.

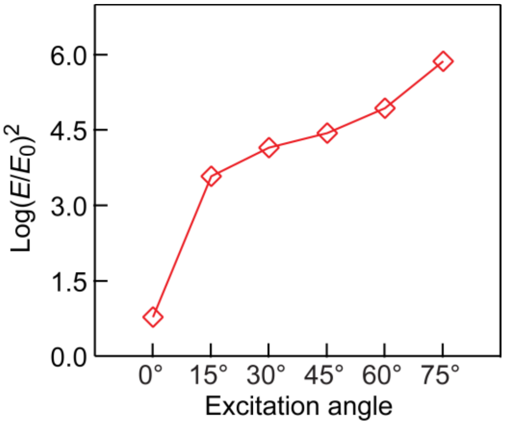

Figure S25. Angle-dependent local electric field intensity enhancement. The field intensity enhancement of the plasmonic nanogap hotspot is plotted as a function of the incidence angle for the (92 NS)@ZIF/film structure. The gap distance is 8.3 nm.

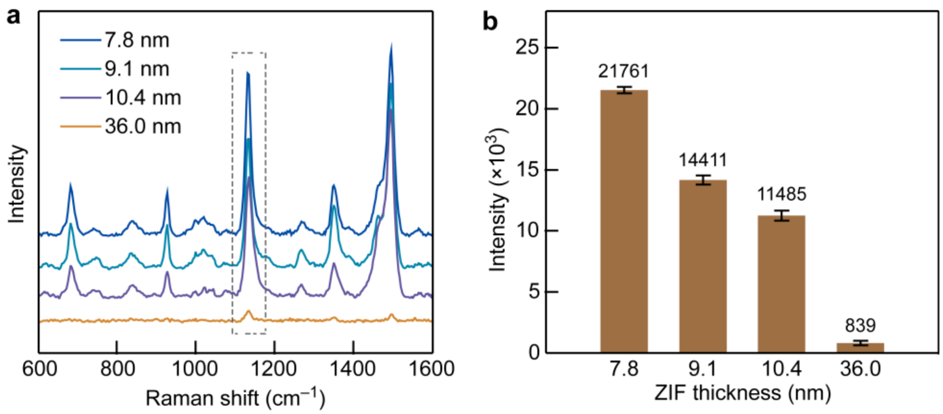

Figure S26. Raman signals of the ZIF shell. (a) SERS spectra. (b) Integrated intensities of the peak indicated with the dashed rectangle in (a). The (92 NS)@ZIF/film structures with different gap distances were measured.

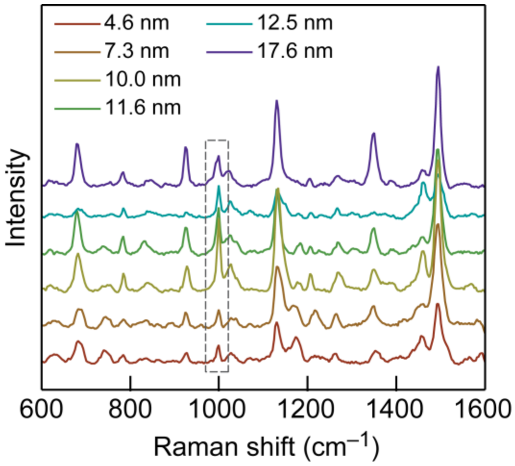

Figure S27. SERS spectra of toluene using the (92 NS)@ZIF/film structures with different gap distances. The concentration of toluene is 2.89×10^7 mg m^{-3}.

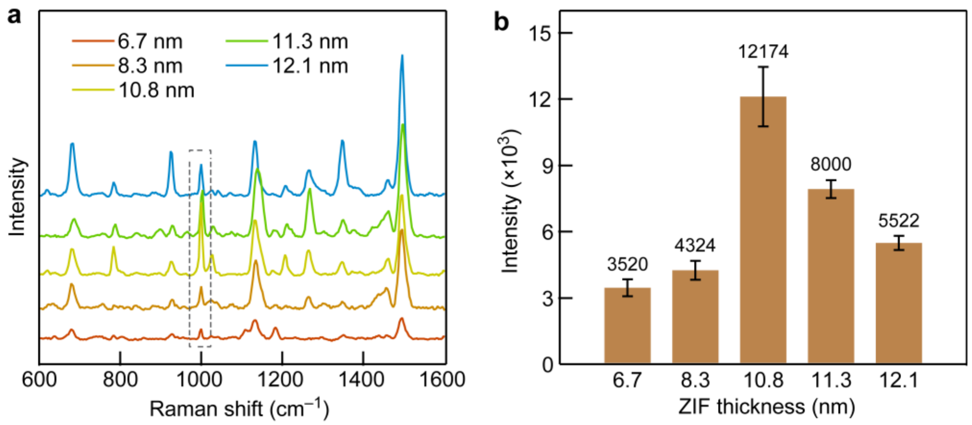

Figure S28. Raman signals of toluene. (a) SERS spectra. (b) Integrated intensities of the peak indicated with the dashed rectangle in (a). The (80 NS)@ZIF/film structures with different gap distances were measured. The concentration of toluene is 2.89×10^7 mg m^{-3}.

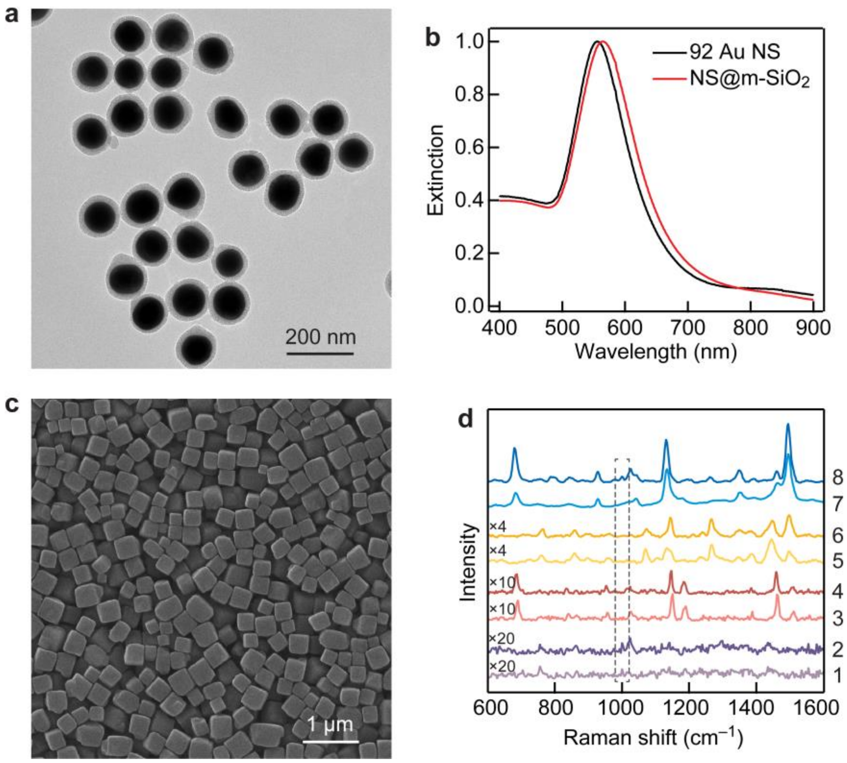

Figure S29. Comparison of the SERS performances with other substrates. (a) TEM image of (92 NS)@(mesoporous SiO_2). The thickness of the mesoporous SiO_2 (m-SiO_2) shell is 10.7 ± 2.4 nm. (b) Extinction spectra of the 92 nm Au NSs and the (92 NS)@m-SiO_2 nanoparticles in water. (c) SEM image of the cubic ZIF nanocrystals with an edge length of 293 ± 40 nm. (d) SERS spectra of toluene using the substrates of the Au-film-supported (92 NS)@m-SiO_2 nanoparticles (1 and 2), ZIF nanocrystals (3 and 4), bare 92 nm Au NSs (5 and 6), and (92 NS)@ZIF nanoparticles (7 and 8), respectively. The curves of 1, 3, 5, and 7 are the SERS signals of the substrates, and the curves of 2, 4, 6, and 8 are the SERS spectra of toluene. The concentration of toluene used for these substrates is 28.9 mg m^{-3}. The characteristic signals of the curves of 5 and 6 can be ascribed to the CTAB molecules adsorbed on the Au NS surface. The 762 cm^{-1} vibrational peak arises from the methyl rocking vibration of the $(CH_3)_3N^+$ group. The peaks at 1071 and 1134 cm^{-1} are caused by the C–C stretching vibrations. The peaks at 1267, 1446, and 1498 cm^{-1} are originated from the twisting, wag vibrations, and bending vibrations of CH_2, respectively.

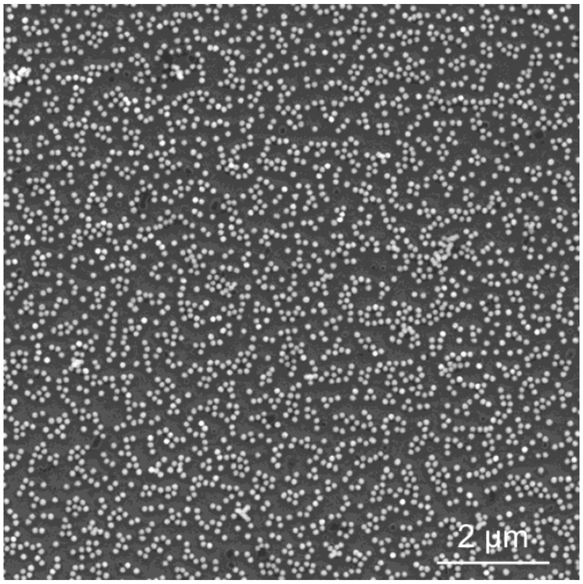

Figure S30. SEM image of the (92 NS)@ZIF nanoparticles supported on a Si substrate.

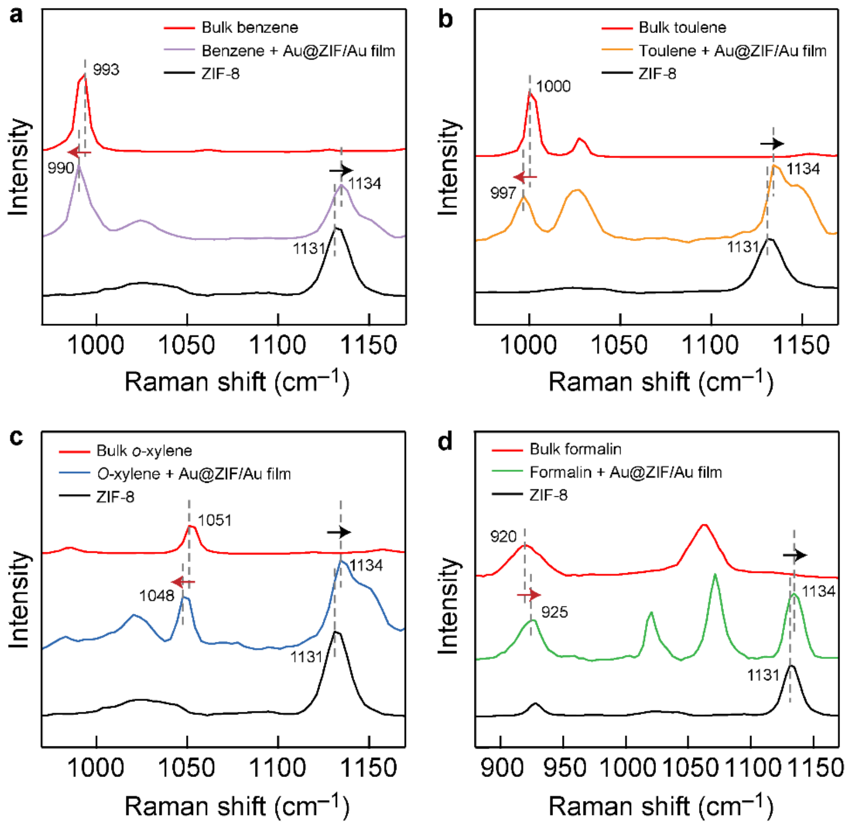

Figure S31. Raman spectra of the VOCs. (a) Benzene. (b) Toluene. (c) *o*-Xylene. (d) Formalin. The Raman spectra were measured in the presence and absence of the NS@ZIF/film substrates, respectively. The intermolecular interactions between ZIF-8 and these target analytes induce

notable shifts in their characteristic vibrational peaks. Specifically, the π-π interactions are implicated in the shifts of the ring-breathing modes: benzene, from 993 to 990 cm^{-1}; toluene, from 1000 to 997 cm^{-1}; and *o*-xylene, from 1051 to 1048 cm^{-1}. In the case of formalin, the C–OH stretching vibration peak shifts from 920 to 925 cm^{-1}, suggesting the influence of hydrogen bonding with ZIF-8. Moreover, the C–N stretching vibrations of the imidazole rings within ZIF-8 exhibit a shift from 1131 to 1134 cm^{-1} in these cases.

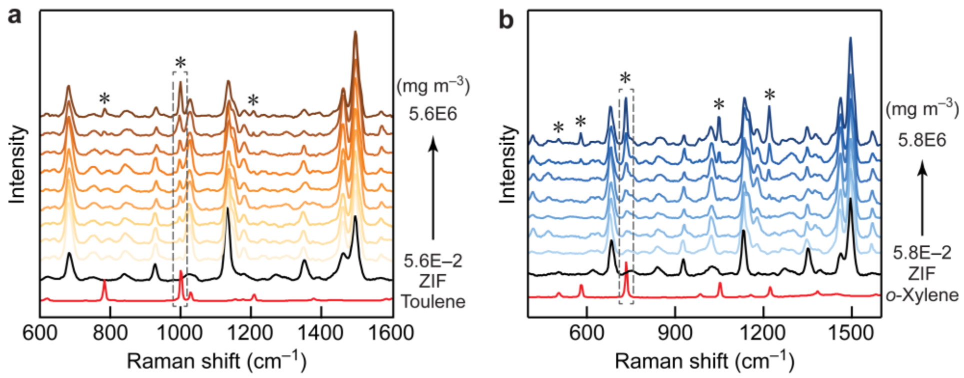

Figure S32. SERS spectra of the two analytes at varying concentrations (mg m^{-3}) using the (92 NS)@ZIF/film structures. (a) Toluene. (b) *O*-Xylene. The gap distance is 9.7 nm. The red curves are the Raman spectra of liquid toluene and *o*-xylene, and the black curves refer to the substrate signals originating from the ZIF shell.

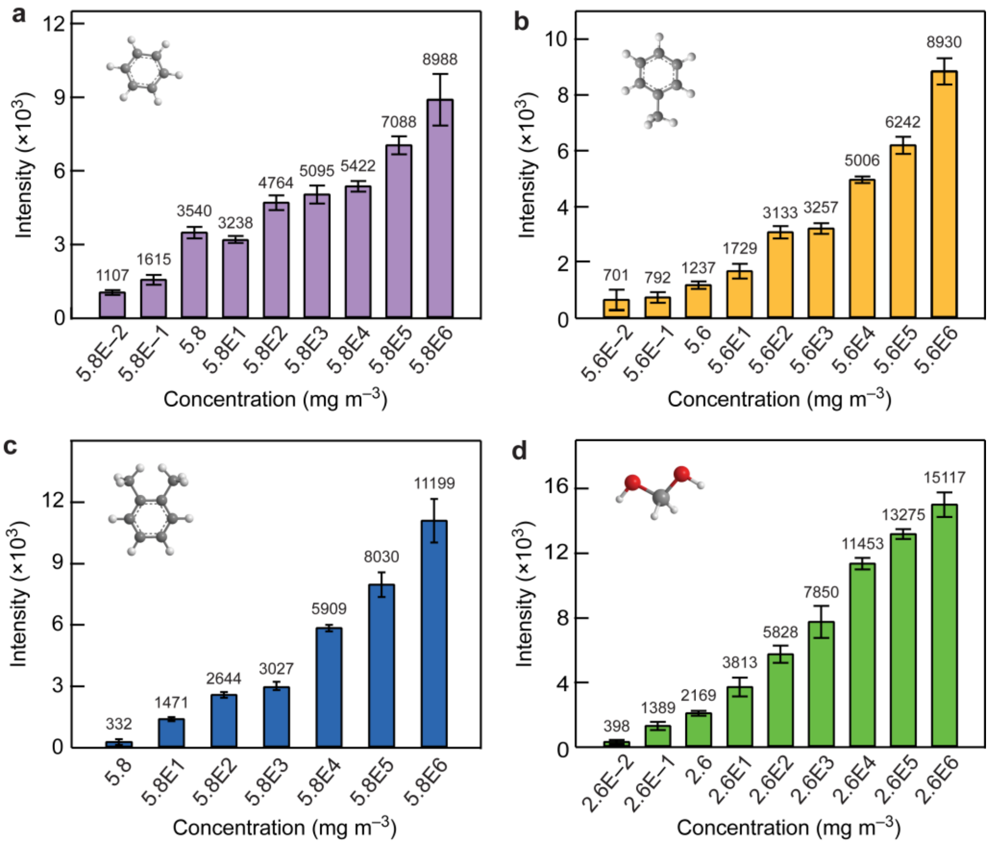

Figure S33. Dependences of the SERS intensities on concentration. (a–d) Integrated intensities of the peaks at 1000 cm^{-1} for benzene, 993 cm^{-1} for toluene, 733 cm^{-1} for *o*-xylene, and 1071 cm^{-1} for formalin, respectively. The error bars were obtained from 20 measured spectra. The insets show the molecular structures of the analytes.

Table S1. Summary of the ZIF Thicknesses, Extinction Wavelengths, and Single-Particle Dark-Field Scattering Wavelengths for the (80 NS)@ZIF and (92 NS)@ZIF Nanoparticles Dispersed in Water and Deposited on the Au Films

	ZIF thickness (nm)	Extinction wavelength in water (nm)	Scattering wavelength on Au films (nm)
(80 NS)@ZIF	4.8 ± 1.7	546	798 ± 12
	6.7 ± 2.7	548	779 ± 16
	8.3 ± 2.1	549	768 ± 15
	11.3 ± 2.6	552	633 ± 16
(92 NS)@ZIF	5.8 ± 1.4	560	>850
	7.8 ± 2.0	562	819 ± 16
	12.5 ± 1.8	567	726 ± 15
	20.3 ± 4.4	572	622 ± 17

The standard deviations for the scattering wavelengths result from the distributions of the ZIF thicknesses and the roughness of the Au film surface. The root-mean-square roughness of the Au films was measured to be 1.7 nm by atomic force microscopy, as reported previously by us.[18]

REFERENCES

(1) Fang, C. H.; Jia, H. L.; Chang, S.; Ruan, Q. F.; Wang, P.; Chen, T.; Wang, J. F. (Gold Core)/(Titania Shell) Nanostructures for Plasmon-Enhanced Photon Harvesting and Generation of Reactive Oxygen Species. *Energy Environ. Sci.* **2014**, *7*, 3431–3438.

(2) Qin, F.; Zhao, T.; Jiang, R. B.; Ruan, Q. F.; Wang, J. F.; Sun, L.-D.; Yan, C.-H.; Lin, H.-Q. Thickness Control Produces Gold Nanoplates with Their Plasmon in the Visible and Near-Infrared Regions. *Adv. Opt. Mater.* **2016**, *4*, 76–85.

(3) Xin, X.; Zhang, Y. Y.; Zhang, L. J.; Zheng, J. G.; Huang, Y. Z.; Fa, H. B. Plasmon-Driven Interfacial Catalytic Reactions in Plasmonic MOF Nanoparticles. *Anal. Chem.* **2021**, *93*, 13219−13225.

(4) Mao, J. J.; Ge, M. Z.; Huang, J. Y.; Lai, Y. K.; Lin, C. J.; Zhang, K. Q.; Meng, K.; Tang, Y. X. Constructing Multifunctional MOF@rGO Hydro-/Aerogels by the Self-Assembly Process for Customized Water Remediation. *J. Mater. Chem. A* **2017**, *5*, 11873–11881.

(5) Hashimoto, K.; Badarla, V.; Kawai1, A.; Ideguchi, T. Complementary Vibrational Spectroscopy. *Nat. Commun.* **2019**, *10*, 4411.

(6) Sakamoto, A.; Tasumi, M. Symmetry of the Benzene Ring and Its Normal Vibrations: The "Breathing" Mode Is Not Always a Normal Vibration of a Benzene Ring. *J. Raman Spectrosc.* **2021**, *52*, 2282–2291.

(7) Hudry, D.; Busko, D.; Popescu, R.; Gerthsen, D.; Howard, I. A.; Richards, B. S. An Enhanced Energy Migration Strategy in Upconverting Nanocrystals: Color-Tuning with High Quantum Yield. *J. Mater. Chem. C* **2019**, *7*, 7371–7377.

(8) Phan-Quang, G. C.; Yang, N. C.; Lee, H. K.; Sim, H. Y. F.; Koh, C. S. L.; Kao, Y.-C.; Wong, Z. C.; Tan, E. K. M.; Miao, Y.-E.; Fan, W.; Liu, T. X.; Phang, I. Y.; Ling, X. Y. Tracking Airborne Molecules from Afar: Three-Dimensional Metal–Organic Framework Surface-Enhanced Raman Scattering Platform for Stand-Off and Real-Time Atmospheric Monitoring. *ACS Nano* **2019**, *13*, 12090–12099.

(9) Moreau, J.; Rinnert, E. Fast Identification and Quantification of BTEX Coupling by Raman Spectrometry and Chemometrics. *Analyst* **2015**, *140*, 3535–3542.

(10) Takahashi, Y.; Shishido, T.; Yamamoto, K.; Sawaji, Y.; Nishida, J.; Pezzotti, G. Do Formalin Fixation and Freeze-Thaw Affect Near-Infrared Raman Spectroscopy of Cartilaginous Tissue? A Preliminary *Ex Vivo* Analysis of Native Human Articular Cartilage. *J. Raman Spectrosc.* **2015**, *46*, 1166–1172.

(11) Hojati, S. F.; Zeinali, T.; Nematdoust, Z. A Novel Method for Synthesis of Bis(indolyl)methanes Using 1,3-Dibromo-5,5-dimethylhydantoin as a Highly Efficient Catalyst under Solvent-Free Conditions. *Bull. Korean Chem. Soc.* **2013**, *34*, 117–120.

(12) Fu, J.-H.; Zhong, Z.; Xie, D.; Guo, Y.-J.; Kong, D.-X.; Zhao, Z.-X.; Zhao, Z.-X.; Li, M. SERS-Active MIL-100(Fe) Sensory Array for Ultrasensitive and Multiplex Detection of VOCs. *Angew. Chem. Int. Ed.* **2020**, *59*, 20489–20498.

(13) Chu, F. C.; Zheng, Y.; Wen, B. Y.; Zhou, L.; Yan, J.; Chen, Y. L. Adsorption of Toluene with Water on Zeolitic Imidazolate Framework-8/Graphene Oxide Hybrid Nanocomposites in a Humid Atmosphere. *RSC Adv.* **2018**, *8*, 2426–2432.

(14) Zhu, X. Z.; Jia, H. L.; Zhu, X. -M.; Cheng, S.; Zhuo, X. L.; Qin, F.; Yang, Z.; Wang, J. F. Selective Pd Deposition on Au Nanobipyramids and Pd Site-Dependent Plasmonic Photocatalytic Activity. *Adv. Funct. Mater.* **2017**, *27*, 1700016.

(15) Zheng, G. C.; Marchi, S.; López-Puente, V.; Sentosun, K.; Polavarapu, L.; Pérez-Juste, I.; Hill, E. H.; Bals, S.; Liz-Marzán, L. M.; Pastoriza-Santos, I.; Pérez-Juste, J. Encapsulation of Single Plasmonic Nanoparticles within ZIF-8 and SERS Analysis of the MOF Flexibility. *Small* **2016**, *12*, 3935–3943.

(16) Pan, Y. C.; Heryadi, D.; Zhou, F.; Zhao, L.; Lestari, G.; Su, H.; Lai, Z. P. Tuning the Crystal Morphology and Size of Zeolitic Imidazolate Framework-8 in Aqueous Solution by Surfactants. *CrystEngComm* **2011**, *13*, 6937–6940.

(17) Chen, H. J.; Shao, L.; Li, Q.; Wang, J. F. Gold Nanorods and Their Plasmonic Properties. *Chem. Soc. Rev.* **2013**, *42*, 2679–2724.

(18) Chow, T. H.; Lai, Y. H.; Lu, W. Z.; Li, N. N.; Wang, J. F. Substrate-Enabled Plasmonic Color Switching with Colloidal Gold Nanorings. *ACS Mater. Lett.* **2020**, *2*, 744−753.
